# A new asymmetric extended family: Properties and estimation methods with actuarial applications

**DOI:** 10.1371/journal.pone.0275001

**Published:** 2022-10-06

**Authors:** Hassan M. Aljohani, Sarah A. Bandar, Hazem Al-Mofleh, Zubair Ahmad, M. El-Morshedy, Ahmed Z. Afify

**Affiliations:** 1 Department of Mathematics & Statistics, College of Science, Taif University, Taif, Saudi Arabia; 2 Department of Mathematics, College of Education, Misan University, Amarah, Iraq; 3 Department of Mathematics, Tafila Technical University, Tafila, Jordan; 4 Department of Statistics, Yazd University, Yazd, Iran; 5 Department of Mathematics, College of Science and Humanities in Al-Kharj, Prince Sattam bin Abdulaziz University, Al-Kharj, Saudi Arabia; 6 Department of Statistics, Faculty of Science, Mansoura University, Mansoura, Egypt; 7 Department of Statistics, Mathematics and Insurance, Benha University, Benha, Egypt; University of Health and Allied Sciences, GHANA

## Abstract

In the present work, a class of distributions, called new extended family of heavy-tailed distributions is introduced. The special sub-models of the introduced family provide unimodal, bimodal, symmetric, and asymmetric density shapes. A special sub-model of the new family, called the new extended heavy-tailed Weibull (NEHTW) distribution, is studied in more detail. The NEHTW parameters have been estimated via eight classical estimation procedures. The performance of these methods have been explored using detailed simulation results which have been ordered, using partial and overall ranks, to determine the best estimation method. Two important risk measures are derived for the NEHTW distribution. To prove the usefulness of the two actuarial measures in financial sciences, a simulation study is conducted. Finally, the flexibility and importance of the NEHTW model are illustrated empirically using two real-life insurance data sets. Based on our study, we observe that the NEHTW distribution may be a good candidate for modeling financial and actuarial sciences data.

## 1 Introduction

Probability distributions have been extensively used to model real-life data is several applied areas. For example, glass fiber, breaking stress of carbon fiber, and gauge lengths data [1] and wind speed data [2]. Speaking broadly, the heavy-tailed models play a vital role in analyzing and modelling data in several applied areas such as banking, risk management, financial and actuarial sciences, and economic, among others. The quality of statistical procedures mainly depends on the considered probability model of the studied phenomenon.

Usually the insurance and financial data sets are positive or right skewed [3, 4], or unimodal shaped [5], and having heavy tails [6]. [7] showed that right-skewed data sets can be modeled using skewed distributions. Therefore, some unimodal and right-skewed distributions were employed to analyze such data sets (see, e.g. [8–10]). The recent developments have been suggested using several approaches including (i) composition of two or more distributions, (ii) transformation of variables and (iii) compounding of distributions. More details can be explored in [11].

Some notable recent proposed families in the statistical literature include the odd Chen-G [12], alpha-power Marshall–Olkin-G [13], type-I half-logistic Weibull-G [14], new flexible generalized class [15], additive odd-G [16], Marshall–Olkin–Weibull-H families [17].

In this paper a flexible class of distributions, called the new extended heavy-tailed-F (NEHT-F) family is studied. The new extended heavy-tailed Weibull (NEHTW) distribution is studied in detail as a special case of this family. The proposed NEHTW model has greater flexibility and it can provide adequate fits in modelling heavy-tailed real data encountered in insurance field and other applied fields. The NEHTW parameters are estimated using eight different estimation methods. The simulation results are conducted to evaluate the performance of these methods of estimation and to choose the best estimation method for the NEHTW parameters based on ranks.

The proposed NEHT-F family are motivated by the following desirable properties. (i) Its special models can provide right-skewed, unimodal, and reversed-J densities which are suitable for modelling heavy-tailed real data encountered in insurance science and many other applied sciences. Furthermore, the hazard rate function (hrf) of the special models of the NEHT-F family can be constant, increasing, modified bathtub, decreasing, J-shaped, and reversed-J shaped hazard rates; (ii) The density functions of its special models can be bimodal, unimodal, symmetric and asymmetric shapes; (iii) The cdf, pdf, hrf of the proposed family have simple closed form expressions. Hence the special sub-models of the NEHT-F family can be used in analyzing and modelling censored data; (iv) The NEHTW distribution, as a special sub-model of NEHT-F family, includes the Weibull distribution as a special case and it can provide adequate fit for heavy-tailed insurance data; (v) We discussed and derived two important risk measure, VaR and TVaR, for the NEHTW model and validate them based on simulations and real data applications, showing that the NEHTW has a heavier tail than the Weibull distribution.

Furthermore, the NEHTW parameters are estimated via eight classical estimation procedures. The performance of these approaches are explored using detailed simulation results which have been ordered, using partial and overall ranks, to determine the best estimation method. This will develop a guideline for choosing the best estimation method for the NEHTW distribution, which we think would be of interest to engineers.

The rest of this work was presented as follows. The NEHT-F family is introduced in Section 2. Some special sub-models of the NEHT-F family are provided in Section 3. The expressions for VaR and TVaR of the NEHTW model along with a simulation study are discussed in Section 4. In Section 5, some mathematical properties of the NEHT-F family are reported. The maximum likelihood estimators of the new family parameters are obtained in Section 6. Eight different classical estimators are discussed to estimate the NEHTW parameters in Section 7. In Section 8, the performance of the introduced estimators is evaluated via Monte Carlo simulations. Two heavy-tailed real-life data sets from the insurance sciences are analysed in Section 9. Some conclusions are provided in Section 10.

## 2 Genesis of the proposed family

The heavy-tailed distributions have heavier right-tails than those of the exponential one. That is, for a baseline *G*(*x*), cumulative distribution function (cdf), the following formula holds

$$\begin{array}{c} \lim_{x\to \infty }e^{px}(1-G(x))=\infty ,\;\;\;\;\;\;\;\;\;\;\;\;p>0. \end{array}$$ (1)

The interested readers can refer to [18–20].

In the present work, an alternative approach to (1), we suggest a empirical approach to find heavy-tailed distributions. The proposed approach is based on the Monte Carlo simulation of the actuarial measures, namely value at risk (VaR) and tail value at risk (TVaR).

Let *X* represent the profit and loss distribution (profit positive and loss negative). The VaR at level *q* ∈ (0, 1) is the lowest number, where the probability that *Y* = −*X* does not exceed *y* is at least 1 − *q*. Mathematically, *VaR*_*q*_(*X*) is the (1 − *q*)^*th*^ quantile of *Y*, i.e.,

$$\begin{array}{c} VaR_{q}(X)=-\text{inf}\{x\in R:F_{X}(x)\}=F_{Y}^{-1}(1-q), \end{array}$$ (2)

for detail see [21, 22].

The TVaR is also referred to as tail conditional expectation (TCE). The TVaR determines the expected value of the losses under the condition that an event outside a certain probability level was occurred.

The TVaR is specified by

$$\begin{array}{c} TVaR_{q}(X)=E[X|X\leq -VaR_{q}(X)]=E[X|X\leq x^{q}], \end{array}$$ (3)

where *x*^*q*^ is the upper quantile given by $x^{q}=\text{inf}\{x\in R:\text{Pr}(X\leq x)>q\},$ for some details see e.g., [21, 23–25].

To apply the simulation approach based on (2) and (3), we first introduce a new family of distributions and then provide a simulation study of the VaR and TVaR for a special sub-model of the proposed family to show empirically the heaviness of the tail of the proposed distribution.

Recently, [26] proposed a new wider family called extended Cordeiro and de Castro (ECC) family which is defined by the cdf

$$\begin{array}{c} G(x;\theta ,\eta ,p,\xi )=1-{\left[\frac{1-F{\left(x;\xi \right)}^{\theta }}{1-pF{\left(x;\xi \right)}^{\theta }}\right]}^{\eta },\;\;\;\;\;\;\;\;\;\;\;\;x\in R, \end{array}$$ (4)

where $\theta ,\eta >0,\xi \in R^{n},$ and *p* ∈ (0, 1). In the ECC family, the space of the parameter *p* is restricted to (0,1), hence the family with cdf (4) can not have enough flexibility to counter complex forms of data. Furthermore, the estimation of parameters of the special models of the ECC family as well as the computation of several distributional characteristics become very difficult due to the three additional parameters of the ECC family. Therefore, in this paper a flexible class of distributions, called the NEHT-F family is studied. The new family is proposed by keeping constant *θ* = 1 in (4) (to reduce the number of parameters), and instead of parameter *p*, we use a new parameter *σ* > 0 with unbounded upper bound unlike the upper limit of *p*. The new extended class of heavy-tailed distributions is introduced via the T-*X* family approach of [27].

The T-*X* family approach is specified by the cdf

$$\begin{array}{c} G(x)=\int_{a_{1}}^{V[F(x;\xi )]}\;\;w(t)\;\;dt,\;\;\;\;\;\;\;\;\;\;\;x\in R, \end{array}$$ (5)

where *V*[*F*(*x*;***ξ***)] satisfies three conditions as illustrated in [27].

The pdf corresponding to (5) is

$$g(x)=\{\frac{\partial }{\partial x}V[F(x;\xi )]\}\;\;w\{V[F(x;\xi )]\},\;\;\;\;\;x\in R.$$

Let *w*(*t*) be the exponential density, i.e., *w*(*t*) = *ηe*^−*ηt*^. Then, we replace the upper limit of (5) by $V[F(x;\xi )]=-\text{log}\{\frac{1-F(x;\xi )}{1-\bar{\sigma }F(x;\xi )}\}$, we obtain the cdf of the proposed family. If a random variable (*rv*) *X* follows the proposed NEHT-F family, then its cdf takes the form

$$\begin{array}{c} G(x;\eta ,\sigma ,\xi )=1-{\left\{\frac{1-F(x;\xi )}{1-\bar{\sigma }F(x;\xi )}\right\}}^{\eta },\;\;\;\;\;\;\;\;\;\;\;\;\;\;\;\;\;\;\;\;\sigma ,\eta >0,\;x,\xi \in R^{n}, \end{array}$$ (6)

where $\bar{\sigma }=(1-\sigma )$ and *F*(*x*; ***ξ***) is the baseline cdf with a parameter vector, $\xi \in R^{n}.$ It is important to note that if *σ* is restricted to (0,1), then (6) can be considered as a special case of (4).

The pdf corresponding to (6) is

$$\begin{array}{c} g(x;\eta ,\sigma ,\xi )=\eta \sigma f(x;\xi )\frac{{\left\{1-F(x;\xi )\right\}}^{\eta -1}}{{\left\{1-\bar{\sigma }F(x;\xi )\right\}}^{\eta +1}},\;\;\;\;\;\;\;\;x\in R. \end{array}$$ (7)

## 3 Sub-models description

This section provides some sub-models from NEHT-F family called the NEHTW, NEHT-normal (NEHTN), and NEHTW-gamma (NEHTG) distributions. These sub-models can provide bimodal, unimodal, symmetric and asymmetric density shapes, and constant, increasing, modified bathtub, decreasing, J-shaped, and reversed-J shaped hazard rate functions.

### 3.1 NEHTW distribution

Consider the *rv* *X* that follows the one-parameter Weibull distribution with cdf $F\left(x;\alpha \right)=1-e^{-x^{\alpha }},\;\;\;x\geq 0,\;\alpha >0$, and pdf $f\left(x;\alpha \right)=\alpha x^{\alpha -1}e^{-x^{\alpha }}$. Then, the cdf of the NEHTW distribution takes the form

$$\begin{array}{c} G(x;\eta ,\sigma ,\alpha )=1-{\left\{\frac{e^{-x^{\alpha }}}{1-\bar{\sigma }(1-e^{-x^{\alpha }})}\right\}}^{\eta },\;\;\;\;\;\;\;\;\;x>0,\;\;\alpha ,\sigma ,\eta >0. \end{array}$$ (8)

The corresponding pdf and hrf of the NEHTW model are

$$\begin{array}{c} g(x;\eta ,\sigma ,\alpha )=\frac{\alpha \sigma \eta x^{\alpha -1}e^{-\eta x^{\alpha }}}{{\left\{1-\bar{\sigma }(1-e^{-x^{\alpha }})\right\}}^{\eta +1}},\;\;\;\;\;\;\;\;\;\;x>0 \end{array}$$ (9)

and

$$\begin{array}{c} h(x;\eta ,\sigma ,\alpha )=\frac{\alpha \sigma \eta x^{\alpha -1}}{\left\{1-\bar{\sigma }(1-e^{-x^{\alpha }})\right\}},\;\;\;\;\;\;\;\;\;\;x>0, \end{array}$$ (10)

The QF of the NEHTW model is given by

$$\begin{array}{c} Q_{X}\left(p\right)={\left[\text{log}(\frac{{\left(1-p\right)}^{-1/\eta }-1}{\sigma }+1)\right]}^{\frac{1}{\alpha }}, \end{array}$$ (11)

where 0 < *p* < 1.

The one parameter Weibull distribution with parameter *α* follows as a special sub-model from the NEHTW distribution when *η* = *σ* = 1. For *σ* = 1, the two-parameter Weibull distribution follows as a special sub-model from the NEHTW model. The exponential distribution follows as a special case for *α* = *σ* = 1. For *σ* = 1 and *α* = 2, the NEHTW model reduces to the Rayleigh distribution.

**Fig 1:**
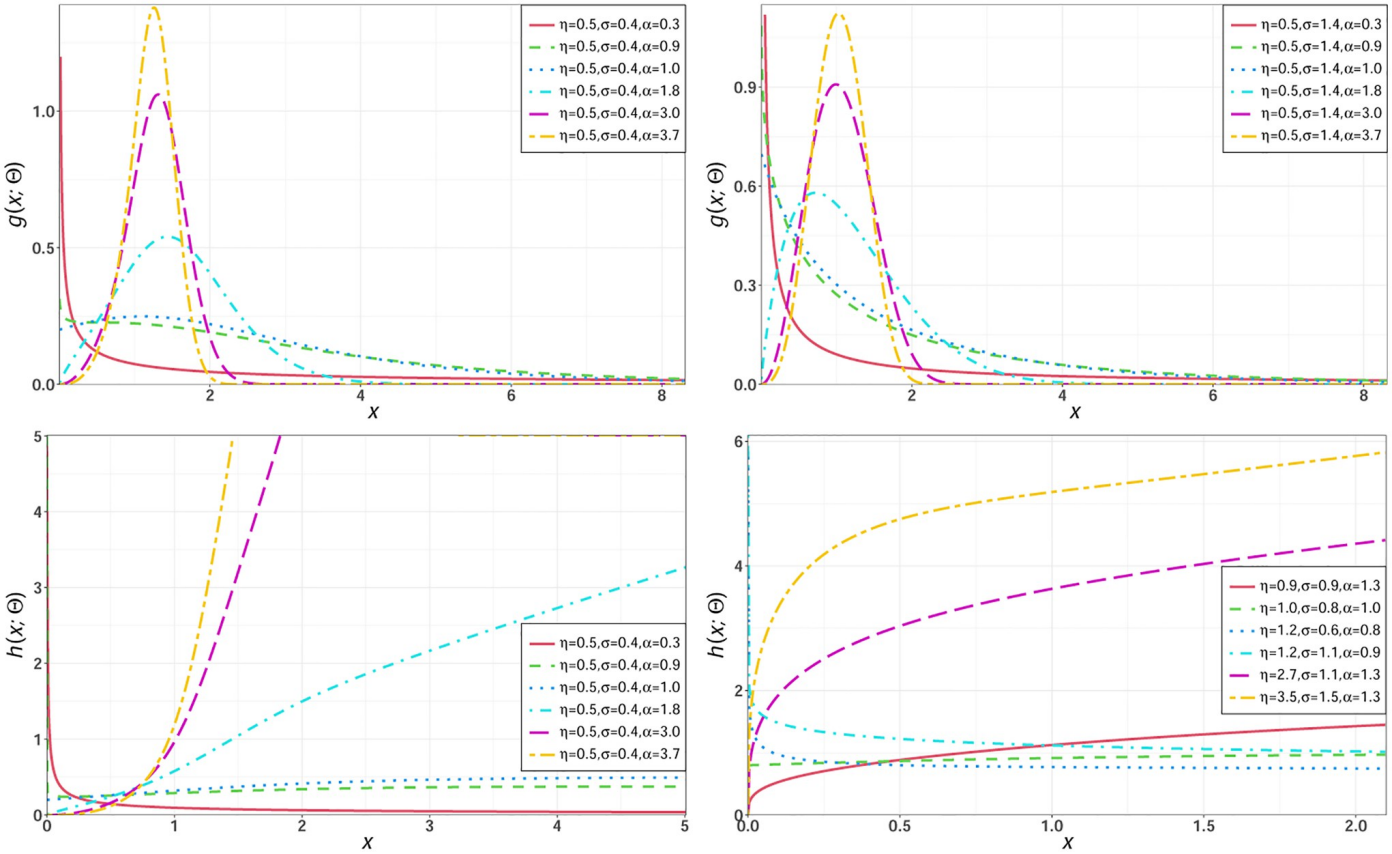
Different shapes for the pdf and hrf of the NEHTW model.

For selected parameters values of the NEHTW distribution, some possible shapes for its density and hazard functions are sketched in Fig 1.

### 3.2 NEHTN distribution

Consider the *rv* *X* that follows the normal distribution with cdf $F\left(x;\mu ,\sigma \right)=\Phi \left(\frac{x-\mu }{\sigma }\right)$ where $\Phi \left(y\right)=\frac{1}{\sqrt{2\pi }}\int_{-\infty }^{y}e^{-\frac{1}{2}x^{2}}dx$, and pdf $f\left(x;\mu ,\sigma \right)=\frac{1}{\sigma }\phi \left(\frac{x-\mu }{\sigma }\right)$ where x∈R,μ∈R,σ>0 and $\phi \left(t\right)=\frac{1}{\sqrt{2\pi }}e^{-\frac{1}{2}x^{2}}$. Then, the cdf of the NEHTN distribution takes the form

$$\begin{array}{c} G(x;\eta ,\sigma_{1},\mu ,\sigma_{2})=1-{\left\{\frac{1-\Phi (\frac{x-\mu }{\sigma_{2}})}{1-\bar{\sigma_{1}}\Phi \left(\frac{x-\mu }{\sigma_{2}}\right)}\right\}}^{\eta },\;\;\;\;\;\;\;\;\;x\in R,\;\;\sigma_{1},\sigma_{2},\eta >0,\mu \in R. \end{array}$$ (12)

The corresponding pdf and hrf of the NEHTN model are

$$\begin{array}{c} g(x;\eta ,\sigma_{1},\mu ,\sigma_{2})=\frac{\eta \sigma_{1}}{\sigma_{2}}\phi (\frac{x-\mu }{\sigma_{2}})\frac{{\left\{1-\Phi (\frac{x-\mu }{\sigma_{2}})\right\}}^{\eta -1}}{{\left\{1-\bar{\sigma_{1}}\Phi \left(\frac{x-\mu }{\sigma_{2}}\right)\right\}}^{\eta +1}},\;\;\;\;\;\;\;\;\;\;x\in R \end{array}$$ (13)

and

$$\begin{array}{c} h(x;\eta ,\sigma ,\alpha )=\frac{\eta \sigma_{1}}{\sigma_{2}}\frac{\phi (\frac{x-\mu }{\sigma_{2}})}{\left\{1-\Phi (\frac{x-\mu }{\sigma_{2}})\}\{1-\bar{\sigma_{1}}\Phi \left(\frac{x-\mu }{\sigma_{2}}\right)\right\}},\;\;\;\;\;\;\;\;\;\;x\in R. \end{array}$$ (14)

**Fig 2:**
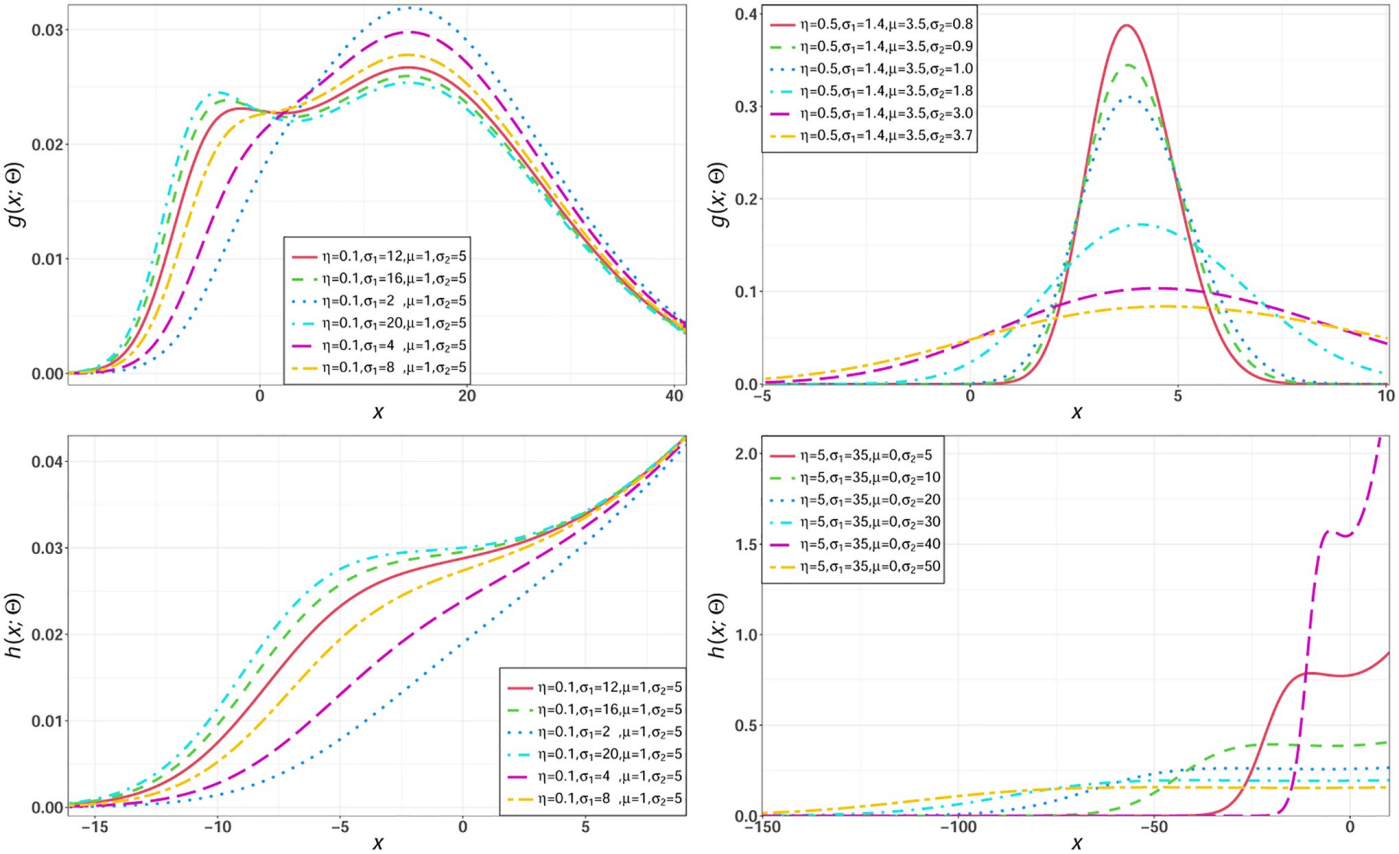
Different shapes for the pdf and hrf of the NEHTN model.

For selected parameters values of the NEHTN distribution, some possible shapes for its density and hazard functions are sketched in Fig 2, the shape of the NEHTN distribution can be unimodal, bimodal, right skewed or symmetry.

### 3.3 NEHTG distribution

Consider the *rv* *X* that follows the gamma (G) distribution with cdf $F\left(x;\alpha ,\beta \right)=\frac{1}{\Gamma (\alpha )}\gamma (\alpha ,\frac{x}{\beta })$ where $\Gamma (k)=\int_{0}^{\infty }t^{\alpha -1}e^{-t}dt$ is the G function and $\gamma (k,y)=\int_{0}^{y}t^{k-1}e^{-t}dt$ is the lower incomplete G function, and pdf $f\left(x;\alpha ,\beta \right)=\frac{1}{\Gamma \left(\alpha \right)\beta^{\alpha }}x^{\alpha -1}e^{-\frac{x}{\beta }}$ where *x* > 0, *α*, *β* > 0. Then, the cdf of the NEHTG distribution takes the form

$$\begin{array}{c} G(x;\eta ,\sigma ,\alpha ,\beta )=1-{\left\{\frac{1-\frac{1}{\Gamma (\alpha )}\gamma (\alpha ,\frac{x}{\beta })}{1-\frac{\bar{\sigma_{1}}}{\Gamma (\alpha )}\gamma (\alpha ,\frac{x}{\beta })}\right\}}^{\eta },\;\;\;\;\;\;\;\;\;x>0,\;\;\alpha ,\beta ,\sigma ,\eta >0. \end{array}$$ (15)

The corresponding pdf and hrf of the NEHTG model are

$$\begin{array}{c} g(x;\eta ,\sigma ,\alpha ,\beta )=\frac{\eta \sigma }{\Gamma \left(\alpha \right)\beta^{\alpha }}x^{\alpha -1}e^{-\frac{x}{\beta }}\frac{{\left\{1-\frac{1}{\Gamma (\alpha )}\gamma (\alpha ,\frac{x}{\beta })\right\}}^{\eta -1}}{{\left\{1-\frac{\bar{\sigma }}{\Gamma (\alpha )}\gamma (\alpha ,\frac{x}{\beta })\right\}}^{\eta +1}},\;\;\;\;\;\;\;\;x>0 \end{array}$$ (16)

and

$$\begin{array}{c} h(x;\eta ,\sigma ,\alpha )=\frac{\eta \sigma }{\Gamma \left(\alpha \right)\beta^{\alpha }}\frac{x^{\alpha -1}e^{-\frac{x}{\beta }}}{\left\{1-\frac{1}{\Gamma (\alpha )}\gamma (\alpha ,\frac{x}{\beta })\right\}\left\{1-\frac{\bar{\sigma }}{\Gamma (\alpha )}\gamma (\alpha ,\frac{x}{\beta })\right\}},\;\;\;\;\;\;\;\;\;\;x>0. \end{array}$$ (17)

**Fig 3:**
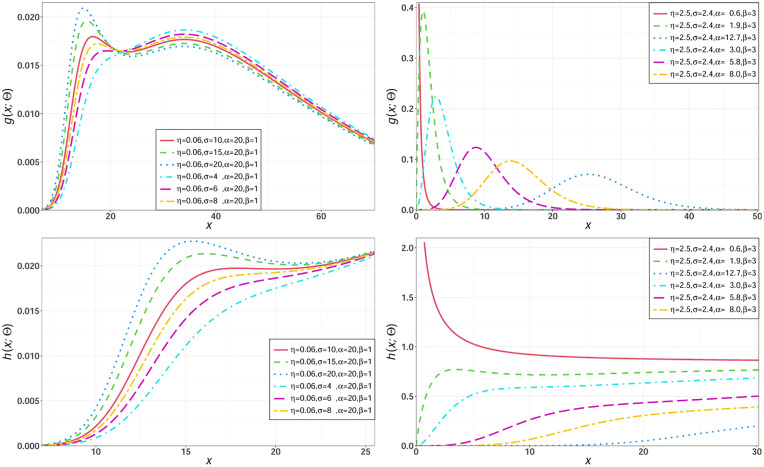
Different shapes for the pdf and hrf of the NEHTG model.

For selected parameters values of the NEHTG distribution, some possible shapes for its density and hazard functions are sketched in Fig 3, the shape of the NEHTG distribution can be unimodal, bimodal, right skewed, symmetry or decreasing.

## 4 Two risk measures and numerical results

The actuaries are often interested in evaluating the exposure to market risks appear due to changes in some underlying variables including prices of equity, exchange rates or interest rates. This section is devoted to determining two important risk measures namely, VaR and TVaR of the proposed NEHTW distribution, which are useful in portfolio optimization.

The VaR is known in actuarial sciences as the quantile premium principle or quantile risk measure and it has been adopted by practitioners as a standard financial market risk. The VaR of a *rv* *X* is defined by the *qth* quantile of its cdf and it may be specified with a certain degree of confidence, *q* which is typically can be 90%, 95% or 99%, as shown in [28]. If *X* has cdf (8), then the VaR of *X* is given by

$$\begin{array}{c} x_{q}={\left\{-\text{log}[\frac{{\left(1-q\right)}^{\frac{1}{\eta }}\;\;\left(\bar{\sigma }-1\right)}{\bar{\sigma }\;\;{\left(1-q\right)}^{\frac{1}{\eta }}-1}]\right\}}^{\frac{1}{\alpha }}, \end{array}$$ (18)

where *q* is the *qth* quantile of *X*.

The TVaR is an important measure to determine the expected value losses given that an event outside a certain probability level is occurred. Hence, the TVaR of the NEHTW model is specified by

$$\begin{array}{c} TVaR=\frac{1}{1-q}\int_{VaR_{q}}^{\infty }x\;g(x;\alpha ,\sigma ,\eta )dx. \end{array}$$ (19)

Inserting (9) in Eq (11), we can write

$$TVaR=\frac{1}{1-q}\;\;\int_{VaR_{q}}^{\infty }x\;\frac{\eta \sigma \alpha x^{\alpha -1}e^{-\eta x^{\alpha }}}{{\left\{1-\bar{\sigma }(1-e^{-x^{\alpha }})\right\}}^{\eta +1}}\;\;dx.$$

On solving, we get

$$\begin{array}{c} TVaR=\frac{\sigma \eta }{(1-q){\left(1+\eta \right)}^{\frac{1}{\alpha }+1}}\sum_{i,j=0}^{\infty }{\left(-1\right)}^{j}{\left(\bar{\sigma }\right)}^{j}\left(\begin{array}{c} \eta +i\\ i \end{array}\right)\Gamma \left(\frac{1}{\alpha }+1,\eta \left(j+1\right){\left(VaR_{q}\right)}^{\alpha }\right). \end{array}$$ (20)

In the next section, based on (10) and (19), we provide a simulation study of VaR and TVaR to show empirically the heaviness of the tail of the NEHTW distribution.

In this section, numerical simulation results were presented for the VaR and TVaR of the two-parameter Weibull and the NEHTW distributions for several values of their parameters. The process was described below as follows:

1. Generating random samples of size *n* = 100 from both Weibull and NEHTW distributions.
2. Estimating the parameters of both models by the maximum likelihood approach.
3. The VaR and TVaR of both distributions were calculated using 1000 repetitions.

**Fig 4:**
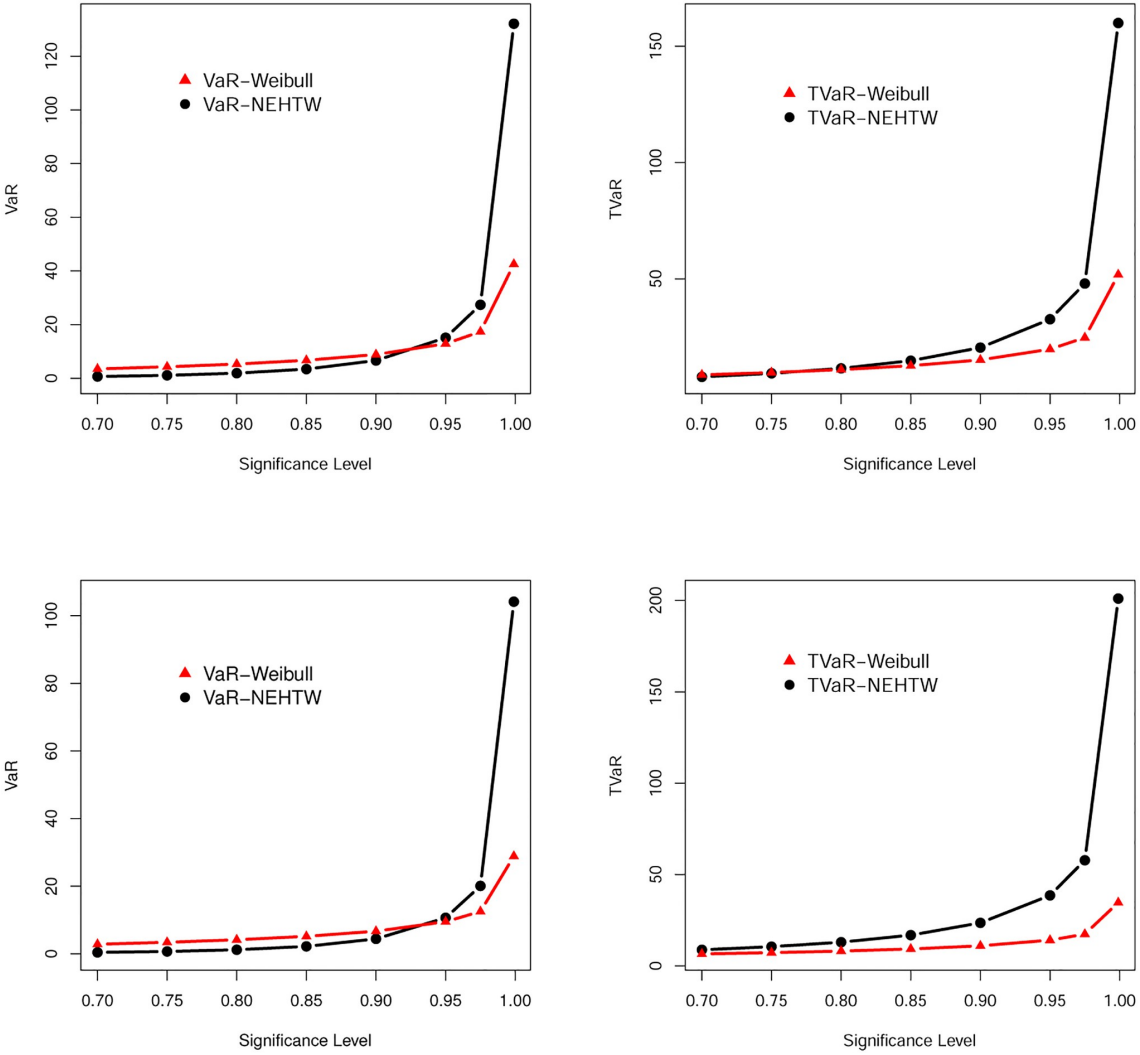
Shapes of the VaR and TVaR of the NEHTW and Weibull distributions based on Tables 1 and 2.

The numerical values of the VaR and TVaR were reported in Tables 1 and 2. For visual comparison of the results in Tables 1 and 2, the graphs for the VaR and TVaR of the Weibull and NEHTW distributions are sketched in Fig 4.

**Table 1:** Simulated values of the VaR and TVaR at different significance levels for *n* = 100.

Dist.	Par	Significance Level	VaR	TVaR
Weibull	*α* = 0.5 *γ* = 0.5	0.700	3.4961	8.7632
		0.750	4.2763	9.7418
		0.800	5.2926	10.9872
		0.850	6.6942	12.6657
		0.900	8.8281	15.1613
		0.950	12.8569	19.7485
		0.975	17.3088	24.7021
		0.999	42.4103	51.6878
NEHTW	*η* = 0.5 *σ* = 3.0 *α* = 0.5	0.700	0.6636	7.9379
		0.750	1.1060	9.4452
		0.800	1.9011	11.6024
		0.850	3.4161	14.9060
		0.900	6.6073	20.5543
		0.950	15.0764	32.7935
		0.975	27.3164	48.1846
		0.999	131.7658	160.5142

**Table 2:** Simulated values of the VaR and TVaR at different significance levels for *n* = 100.

Dist.	Par	Significance Level	VaR	TVaR
Weibull	*α* = 1.2 *γ* = 1.0	0.700	2.8319	6.5988
		0.750	3.4177	7.2958
		0.800	4.1702	8.1756
		0.850	5.1926	9.3500
		0.900	6.7227	11.0761
		0.950	9.5483	14.1990
		0.975	12.6023	17.5152
		0.999	29.08753	34.9514
NEHTW	*η* = 1.0 *σ* = 0.8 *α* = 1.2	0.700	0.4025	8.8077
		0.750	0.6760	10.5085
		0.800	1.1806	12.9734
		0.850	2.1790	16.8181
		0.900	4.3888	23.5508
		0.950	10.6088	38.5356
		0.975	19.9863	57.7103
		0.999	103.4749	200.3137

The model with greater values of both VaR and TVaR is said to have a heavier tail. The simulation values in Tables 1 and 2 illustrate that the NEHTW distribution, with higher values of both VaR and TVaR, has a heavier tail than Weibull distribution. This important result can be shown graphically in Fig 4.

## 5 Properties of the NEHT-G family

Some mathematical quantities of the NEHT-G family were introduced in this section.

### 5.1 Linear representation

Using the two power series

(1+νz)-η=∑j=0∞(-ηj)νjzj (21)

and

(1-z)η=∑k=0∞(-1)k(ηk)zk,|z|<1. (22)

The NEHT-G density can be expressed as

g(x;η,σ,ξ)=ησ∑j,k=0∞(-1)k(-η-1j)(η-1k)(σ-1)jF(x;ξ)j+kf(x;ξ).

Hence, the pdf of the NEHT-G family takes the form

$$\begin{array}{c} g\left(x;\eta ,\sigma ,\xi \right)=\sum_{j,k=0}^{\infty }\delta_{j,k}\;\;h_{\left(j+k+1\right)}\left(x\right), \end{array}$$ (23)

where *h*_(*j*+*k*+1)_(*x*) is the exponentiated-G (exp-G) pdf with positive power parameter (*j* + *k* + 1) and

δj,k=ησ(σ-1)jj+k+1(-1)k(-η-1j)(η-1k).

Hence, the density function of the NEHT-G family can be expressed as a linear combination of exp-G densities. So, Eq (23) reveals that some mathematical quantities of the proposed family can be derived from the quantities of the exp-G family. Hereafter, let *Y*_*j*+*k*+1_ denote a *rv* having the exp-G density with power parameter (*j* + *k* + 1), then some properties of the *rv* *X* are expressed simply from those of *Y*_*j*+*k*+1_.

### 5.2 Moments and generating function

The *n*th moment of the NEHT-G family was expressed from (23) as

$$\begin{array}{c} \mu_{n}^{\prime }=\sum_{j,k=0}^{\infty }\delta_{j,k}\;E\left(Y_{j+k+1}^{n}\right). \end{array}$$

The *r*th incomplete moment of the NEHT-G family follows simply from (23) as

$$\begin{array}{c} m_{r}\left(t\right)=\int_{-\infty }^{t}\;x^{r}\;g\left(x;\eta ,\sigma ,\xi \right)dx=\sum_{j,k=0}^{\infty }\delta_{j,k}\;\int_{-\infty }^{t}\;x^{r}\;h_{j+k+1}\left(x\right)dx. \end{array}$$ (24)

The first incomplete moment (FIM) of *X* follows from (23) as

$$m_{1}\left(t\right)=\sum_{j,k=0}^{\infty }\delta_{j,k}\;P_{j+k+1}\left(t\right),$$

where $P_{j+k+1}\left(t\right)=\int_{-\infty }^{t}\;x\;h_{j+k+1}\left(x\right)dx=\left(j+k+1\right)\;\int_{0}^{G\left(t\right)}\;u^{j+k}Q_{G}\left(u\right)du$ is the FIM of the exp-G family which can be determined numerically or analytically from a baseline qf *Q*_*G*_(*u*) = *G*^−1^(*u*;***ξ***).

The moment generating function (mgf) of the NEHT-G family was derived from (23) as

$$M\left(t\right)=\sum_{j,k=0}^{\infty }\delta_{j,k}\;M_{j+k+1}\left(t\right)=\sum_{j,k=0}^{\infty }\left(j+k+1\right)\;\delta_{j,k}\;\pi \left(t,j+k\right),$$

where *M*_*j*+*k*+1_(*t*) is the mgf of *Y*_*j*+*k*+1_ and $\pi \left(t,j+k\right)=\int_{0}^{1}u^{k+j}\text{exp}[t\;Q_{G}\left(u\right)]du$. Then, *M*(*t*) follows from the exp-G mgf.

### 5.3 Rényi entropy

The Rényi entropy (RE) of a *rv* *X* is a measure of variation of uncertainty. The RE is given by

$$I_{\upsilon }\left(X\right)={\left(1-\upsilon \right)}^{-1}\text{log}\left(\int_{-\infty }^{\infty }g{\left(x\right)}^{\upsilon }dx\right),\;\;\;\upsilon >0\;\text{and}\;\upsilon \neq 1.$$

Using the NEHT-G density, we obtain

$$g{\left(x;\eta ,\sigma ,\xi \right)}^{\upsilon }={\left(\eta \sigma \right)}^{\upsilon }f{\left(x\right)}^{\upsilon }\frac{{\left[1-F\left(x\right)\right]}^{\upsilon \left(\eta -1\right)}}{{\left[1+\left(\sigma -1\right)\;\;F\left(x\right)\right]}^{\upsilon \left(\eta +1\right)}}.$$

By applying the two power series in (21) and (22), we have

g(x)υ=(ησ)υ∑j,k=0∞(-1)k(σ-1)j(-υ(η+1)j)(υ(η-1)k)f(x)υF(x)j+k.

Then, the RE of the NEHT-G family takes the form

$$I_{\upsilon }\left(X\right)={\left(1-\upsilon \right)}^{-1}\;\text{log}\left(\sum_{j,k=0}^{\infty }\vartheta_{j,k}\int_{-\infty }^{\infty }f{\left(x\right)}^{\upsilon }F{\left(x\right)}^{j+k}dx\right),$$

where

ϑj,k=(-1)k(ησ)υ(σ-1)j(-υ(η+1)j)(υ(η-1)k).

### 5.4 Order statistics

Let *X*_1_, …, *X*_*n*_ be a random sample from the NEHT-G family and consider their associated order statistics, *X*_1:*n*_, …, *X*_*n*:*n*_. Then, the pdf of the *s*th order statistic *X*_*s*:*n*_, *g*_*s*:*n*_(*x*), has the form

$$\begin{array}{c} g_{s:n}\left(x\right)=\frac{n!\;\;f\left(x;\xi \right)}{\left(s-1\right)!\left(n-s\right)!}\;{\left[F\left(x\right)\right]}^{s-1}\;{\left[1-F\left(x\right)\right]}^{n-s},\;s=1,2,..,n, \end{array}$$

Using the cdf and pdf of the NEHT-G class, the pdf of the *X*_*s*:*n*_ reduces to

gs:n(x)=n!ησf(x)(s-1)!(n-s)!∑r=0s-1(-1)r(s-1r)[1-F(x)]φ-1[1+(σ-1)F(x)]φ+1,

where *φ* = *η*(*n* + *r* − *s* + 1). Applying the two power series in (21) and (22) to the above equation, we obtain

gs:n(x)=n!ησ(s-1)!(n-s)!∑r=0s-1∑j,k=0∞(-1)r+k(σ-1)-j(s-1r)(-φ-1j)(φ-1k)f(x)F(x)j+k.

Hence, the pdf of the *s*th order statistic for the NEHT-G family is a linear combination of exp-G densities as follows

$$\begin{array}{c} g_{s:n}\left(x\right)=\sum_{j,k=0}^{\infty }\phi_{j,k}\;h_{j+k+1}\left(x\right), \end{array}$$ (25)

where *h*_*j*+*k*+1_(*x*) is the pdf of the exp-G class with power parameter (*j* + *k* + 1) and

ϕj,k=n!ησ(σ-1)j(s-1)!(n-s)!∑r=0s-1(-1)r+k(j+k+1)(s-1r)(-φ-1j)(φ-1k).

Using (25), some mathematical quantities of *X*_*s*:*n*_ follows simply from the properties of *Y*_*j*+*k*+1_.

## 6 Estimations

To estimate the parameters of the NEHT-F family, this section assigns the maximum likelihood estimators (MLEs) to estimating and to provide some simulations to explore the behavior of the MLEs.

### 6.1 Maximum likelihood estimation

Suppose that *x*_1_, *x*_2_, …, *x*_*n*_ are given values of a random sample of size *n* from the NEHT-F family with parameters (*η*, *σ*, ***ξ***). The log–likelihood function takes the form

$$\begin{array}{cc} \ell \left(\Theta \right)= & \;n\;\text{log}\;\eta +n\;\text{log}\;\;\sigma +\sum_{i=0}^{n}\text{log}\;f\left(x_{i};\xi \right)+\left(\eta -1\right)\sum_{i=0}^{n}\text{log}\;\left\{1-F\left(x_{i};\xi \right)\right\}\\ & -\left(\eta +1\right)\sum_{i=0}^{n}\text{log}\;\left\{1-\bar{\sigma }F\left(x_{i};\xi \right)\right\}, \end{array}$$ (26)

where **Θ** = (*η*, *σ*, ***ξ***^⊤^)^⊤^.

The partial derivatives of (26) are

$$\begin{array}{c} \frac{\partial \ell }{\partial \eta }=\frac{n}{\eta }+\sum_{i=0}^{n}\text{log}\left\{1-F\left(x_{i};\xi \right)\right\}-\sum_{i=0}^{n}\text{log}\left\{1-\bar{\sigma }F\left(x_{i};\xi \right)\right\}, \end{array}$$ (27)

$$\begin{array}{c} \frac{\partial \ell }{\partial \sigma }=\frac{n}{\sigma }-\left(\eta +1\right)\sum_{i=0}^{n}\left\{\frac{F\left(x_{i};\xi \right)}{1-\bar{\sigma }F\left(x_{i};\xi \right)}\right\}, \end{array}$$ (28)

and

$$\begin{array}{c} \frac{\partial \ell }{\partial \xi }=\sum_{i=0}^{n}\frac{\partial f\left(x_{i};\xi \right)/\partial \xi }{f\left(x_{i};\xi \right)}-\left(\eta -1\right)\sum_{i=0}^{n}\frac{\partial F\left(x_{i};\xi \right)/\partial \xi }{1-F\left(x_{i};\xi \right)}+\bar{\sigma }\left(\eta +1\right)\sum_{i=0}^{n}\frac{\partial F\left(x_{i};\xi \right)/\partial \xi }{1-\bar{\sigma }F\left(x_{i};\xi \right)}. \end{array}$$ (29)

By setting $\frac{\partial \ell }{\partial \eta }=0$, $\frac{\partial \ell }{\partial \sigma }=0$, and $\frac{\partial \ell }{\partial \xi }=0,$ one can solve them numerically to obtain the MLEs of *η*, *σ* and ***ξ***, respectively.

These nonlinear system of equations can be solved using any statistical software.

## 7 Eight estimation methods for the NEHTW parameters

Eight estimation methods have been opted in this section to estimate the NEHTW parameters, namely: weighted least-squares (WLSE), ordinary least-squares (OLSE), maximum likelihood (MLE), maximum product of spacing (MPSE), Cramér-von Mises (CVME), Anderson-Darling (ADE), right-tail Anderson-Darling (RADE), and percentile estimator (PCE).

Suppose that *x*_1_, *x*_2_, …, *x*_*n*_ are given values of a random sample of size *n* from the NEHTW distribution with parameters *α*, *σ* and *η*.

### 7.1 Maximum likelihood

The log-likelihood function for the NEHTW model in (9) is given by

$$\begin{array}{cc} \ell \left(\Theta \right)= & \;n\text{log}\eta +n\text{log}\;\sigma +n\text{log}\;\alpha +\left(\alpha -1\right)\sum_{i=0}^{n}\text{log}\;\left(x_{i}\right)-\eta \sum_{i=0}^{n}x_{i}^{\alpha }\\ & -\left(\eta +1\right)\sum_{i=0}^{n}\text{log}[1-\left(1-\sigma \right)\left(1-e^{-x_{i}^{\alpha }}\right)], \end{array}$$ (30)

where **Θ** = (*α*, *σ*, *η*)^⊤^. The function provided in (30) can be numerically solved by using Newton-Raphson method (iteration method). The partial derivatives of (30), with respect to the parameters, are

$$\begin{array}{cc} \frac{\partial \ell }{\partial \alpha } & =\frac{n}{\alpha }+\left(\eta +1\right)\left(1-\sigma \right)\sum_{i=1}^{n}\frac{x_{i}^{\alpha }\text{log}\left(x_{i}\right)e^{-x_{i}^{\alpha }}}{1-\left(1-\sigma \right)\left(1-e^{-x_{i}^{\alpha }}\right)}-\eta \sum_{i=1}^{n}x_{i}^{\alpha }\text{log}\left(x_{i}\right)+\sum_{i=1}^{n}\text{log}\left(x_{i}\right), \end{array}$$

$$\begin{array}{c} \frac{\partial \ell }{\partial \eta }=\frac{n}{\eta }-\sum_{i=1}^{n}\text{log}[1-\left(1-\sigma \right)\left(1-e^{-x_{i}^{\alpha }}\right)]-\sum_{i=1}^{n}x_{i}^{\alpha } \end{array}$$

and

$$\begin{array}{c} \frac{\partial \ell }{\partial \sigma }=\frac{n}{\sigma }-\left(\eta +1\right)\sum_{i=1}^{n}\frac{1-e^{-x_{i}^{\alpha }}}{1-\left(1-\sigma \right)\left(1-e^{-x_{i}^{\alpha }}\right)}. \end{array}$$

### 7.2 Ordinary and weighted least-squares

The OLSE of the NEHTW parameters can be obtained by minimizing the following function with respect to *α*, *σ* and *η*,

$$V\left(\alpha ,\sigma ,\eta \right)=\sum_{i=1}^{n}{\left[F\left(x_{i}|\alpha ,\sigma ,\eta \right)-\frac{i}{n+1}\right]}^{2}.$$

Further, the OLSE of the NEHTW parameters can be obtained by solving the non-linear equation

$$\sum_{i=1}^{n}[F\left(x_{i}|\alpha ,\sigma ,\eta \right)-\frac{i}{n+1}]\Delta_{s}\left(x_{i}|\alpha ,\sigma ,\eta \right)=0,\;s=1,2,3,$$

where

$$\begin{array}{c} \Delta_{1}\left(x_{\left(i\right)}|\alpha ,\sigma ,\eta \right)=\frac{\partial }{\partial \beta }F\left(x_{\left(i\right)}|\alpha ,\sigma ,\eta \right),\;\Delta_{2}\left(x_{\left(i\right)}|\alpha ,\sigma ,\eta \right)=\frac{\partial }{\partial \theta }F\left(x_{\left(i\right)}|\alpha ,\sigma ,\eta \right) \end{array}$$

and

$$\begin{array}{ccc} \Delta_{3}\left(x_{\left(i\right)}|\alpha ,\sigma ,\eta \right) & = & \frac{\partial }{\partial a}F\left(x_{\left(i\right)}|\alpha ,\sigma ,\eta \right). \end{array}$$ (31)

The WLSE of the NEHTW parameters are obtained by minimizing the following

$$W\left(\alpha ,\sigma ,\eta \right)=\sum_{i=1}^{n}\frac{{\left(n+1\right)}^{2}\left(n+2\right)}{i\left(n-i+1\right)}{\left[F\left(x_{i}|\alpha ,\sigma ,\eta \right)-\frac{i}{n+1}\right]}^{2},$$

with respect to *α*, *σ* and *η*. Also, the WLSE can be obtained by solving the non-linear equation

$$\sum_{i=1}^{n}\frac{{\left(n+1\right)}^{2}\left(n+2\right)}{i\left(n-i+1\right)}[F\left(x_{i}|\alpha ,\sigma ,\eta \right)-\frac{i}{n+1}]\Delta_{s}\left(x_{i}\right)=0,\;s=1,2,3,$$

where Δ_1_(⋅|*α*, *σ*, *η*), Δ_2_(⋅|*α*, *σ*, *η*) and Δ_3_(⋅|*α*, *σ*, *η*) are defined in Eq (31).

### 7.3 Maximum product of spacing

The MPSE is considered a good alternative to the maximum likelihood method. Let *D*_*i*_(*α*, *σ*, *η*) = *F*(*x*_(*i*)_|*α*, *σ*, *η*) − *F*(*x*_(*i*−1)_|*α*, *σ*, *η*), for *i* = 1, 2, …, *n* + 1, be the uniform spacing of a random sample from the NEHTW model, where *F*(*x*_(0)_|*α*, *σ*, *η*) = 0, *F*(*x*_(*n*+1)_|*α*, *σ*, *η*) = 1 and $\sum_{i=1}^{n+1}D_{i}\left(\alpha ,\sigma ,\eta \right)=1$. The MPSE of the NEHTW parameters can be obtained by maximizing the “geometric mean of the spacing”

$$G\left(\alpha ,\sigma ,\eta \right)={\left[\prod_{i=1}^{n+1}D_{i}\left(\alpha ,\sigma ,\eta \right)\right]}^{\frac{1}{n+1}},$$

with respect to *α*, *σ* and *η*, or by maximizing the “logarithm of the geometric mean” of sample spacings

$$H\left(\alpha ,\sigma ,\eta \right)=\frac{1}{n+1}\sum_{i=1}^{n+1}\text{log}D_{i}\left(\alpha ,\sigma ,\eta \right).$$

Also, the MPSE can be obtained by solving the following nonlinear equations

$$\frac{1}{n+1}\sum_{i=1}^{n+1}\frac{1}{D_{i}\left(\alpha ,\sigma ,\eta \right)}\left[\Delta_{s}\left(x_{\left(i\right)}|\alpha ,\sigma ,\eta \right)-\Delta_{s}\left(x_{\left(i-1\right)}|\alpha ,\sigma ,\eta \right)\right]=0,\;s=1,2,3,$$

where Δ_1_(⋅|*α*, *σ*, *η*), Δ_2_(⋅|*α*, *σ*, *η*) and Δ_3_(⋅|*α*, *σ*, *η*) are defined in Eq (31).

### 7.4 Cramér-von mises

The CVME of the NEHTW parameters are obtained by minimizing

$$C\left(\alpha ,\sigma ,\eta \right)=-\frac{1}{12n}+\sum_{i=1}^{n}{\left[F\left(x_{i}|\alpha ,\sigma ,\eta \right)-\frac{2i-1}{2n}\right]}^{2},$$

with respect to *α*, *σ* and *η*. Also, the CVME can be numerically obtained by solving the following non-linear equation

$$\sum_{i=1}^{n}[F\left(x_{i}|\alpha ,\sigma ,\eta \right)-\frac{2i-1}{2n}]\Delta_{s}\left(x_{i}|\alpha ,\sigma ,\eta \right)=0,\;s=1,2,3,$$

where Δ_1_(⋅|*α*, *σ*, *η*), Δ_2_(⋅|*α*, *σ*, *η*) and Δ_3_(⋅|*α*, *σ*, *η*) are defined in Eq (31).

### 7.5 Anderson-Darling and right-tail Anderson-Darling

Suppose that *x*_(1)_, *x*_(2)_, …, *x*_(*n*)_ is the ordered random sample from *F*(*x*) of the NEHTW model. The ADE of the NEHTW parameters can be obtained by minimizing

$$A\left(\alpha ,\sigma ,\eta \right)=-n-\frac{1}{n}\sum_{i=1}^{n}\left(2i-1\right)\left[\text{log}F\left(x_{i}\right)+\text{log}S\left(x_{i}\right)\right],$$

or by solving the non-linear equation

$$\sum_{i=1}^{n}\left(2i-1\right)[\frac{\Delta_{s}\left(x_{i}\right)}{F\left(x_{i}\right)}-\frac{\Delta_{i}\left(x_{n+1-i}\right)}{S\left(x_{n+1-i}\right)}]=0,\;s=1,2,3,$$

Also, the RADEs of the NEHTW parameters can be obtained by minimizing

$$R\left(\alpha ,\sigma ,\eta \right)=\frac{n}{2}-2\sum_{i=1}^{n}F\left(x_{i:n}|\alpha ,\sigma ,\eta \right)-\frac{1}{n}\sum_{i=1}^{n}\left(2i-1\right)\text{log}S\left(x_{n+1-i:n}|\alpha ,\sigma ,\eta \right),$$

with respect to *β*, *θ* and *a*, which equivalently by solving the non-linear equations

$$-2\sum_{i=1}^{n}\Delta_{s}\left(x_{i:n}|\alpha ,\sigma ,\eta \right)+\frac{1}{n}\sum_{i=1}^{n}\left(2i-1\right)\frac{\Delta_{s}\left(x_{n+1-i:n}|\alpha ,\sigma ,\eta \right)}{S\left(x_{n+1-i:n}|\alpha ,\sigma ,\eta \right)}=0,\;\;s=1,2,3,$$

where Δ_1_(⋅|*α*, *σ*, *η*), Δ_2_(⋅|*α*, *σ*, *η*) and Δ_3_(⋅|*α*, *σ*, *η*) are defined in Eq (31).

### 7.6 Percentile

From (11), the PCE of the parameters of NEHTW model can be obtained by minimizing the following function

$$P\left(\alpha ,\sigma ,\eta \right)=\sum_{i=1}^{n}{\left(x_{\left(i\right)}-{\left[\text{log}\left(\frac{{\left(1-u_{i}\right)}^{-1/\eta }-1}{\sigma }+1\right)\right]}^{\frac{1}{\alpha }}\right)}^{2},$$

with respect to *α*, *σ* and *η*.

## 8 Simulation study

To assessing the performance of the eight estimates aforementioned for the NEHTW parameters, we devote this section.

The simulation results is carried out for the NEHTW distribution by the function optim() with the argument method = “L-BFGS-B” available by **R** software (version 4.0.5) [29]. We generated *N* = 4000 samples of five sizes, where *n* = {50, 80, 120, 200, 300} from the NEHTW distribution for different values of the parameters *η* = {0.40, 1.60, 2.75}, *σ* = {0.50, 1.75, 3.00} and *α* = {0.75, 1.50, 4.00}. The MSEs and biases can be computed, for **Θ** = (*η*, *σ*, *α*)^⊤^, by the formulas: average of absolute value of biases ($\left|Bias\left(\hat{\Theta }\right)\right|$), $|Bias\left(\hat{\Theta }\right)|=$ $\frac{1}{N}\sum_{i=1}^{N}\left|\hat{\Theta }-\Theta \right|$, average of mean square errors (MSEs), $MSEs=\frac{1}{N}\sum_{i=1}^{N}{\left(\hat{\Theta }-\Theta \right)}^{2}$, and average of mean relative errors (MREs), $MREs=\frac{1}{N}\sum_{i=1}^{N}\left|\hat{\Theta }-\Theta \right|/\Theta$.

Using the eight estimators aforementioned with each parameter combination and each sample, the NEHTW parameters are estimated, after that the Bias, MSEs and MREs of the parameter estimates are computed. To save more space, five out of twenty seven simulated outcomes are listed in Tables 3–7, one can easily observe that when the sample size increases, for all parameter combinations except the PCE method. The PCE shows the property of consistency for all parameter combinations, except the combinations **Θ** = (*η* = 0.40, *σ* = 1.75, *α* = 1.50)^⊤^ for the parameter *σ*, and **Θ** = (*η* = 0.40, *σ* = 0.50, *α* = 0.75)^⊤^, **Θ** = (*η* = 0.40, *σ* = 1.75, *α* = 0.75)^⊤^ and **Θ** = (*η* = 0.40, *σ* = 3.00, *α* = 0.75)^⊤^, for the parameter *α*.

**Table 3:** Simulation results for Θ = (*η* = 0.40, *σ* = 0.50, *α* = 0.75)^⊤^.

*n*	Est.	Est. Par.	WLSE	OLSE	MLE	MPSE	CVME	ADE	RADE	PCE
50	|*BIAS*|	$\hat{\eta }$	0.94180^{6}^	1.20390^{7}^	0.72299^{4}^	0.73008^{5}^	1.22850^{8}^	0.71742^{3}^	0.48507^{2}^	0.30437^{1}^
		$\hat{\sigma }$	0.53166^{3}^	0.45695^{1}^	1.16186^{7}^	0.53356^{4}^	0.53922^{5}^	0.52021^{2}^	1.00764^{6}^	1.86642^{8}^
		$\hat{\alpha }$	0.15331^{3}^	0.14843^{2}^	0.19424^{7}^	0.15674^{5}^	0.16001^{6}^	0.14382^{1}^	0.15626^{4}^	0.21878^{8}^
	MSE	$\hat{\alpha }$	0.03539^{3}^	0.03355^{2}^	0.05933^{7}^	0.03598^{4}^	0.03767^{5}^	0.03234^{1}^	0.04283^{6}^	0.06706^{8}^
		$\hat{\sigma }$	1.18220^{4}^	0.69245^{1}^	5.44383^{7}^	1.56205^{5}^	1.04541^{2}^	1.11182^{3}^	4.87864^{6}^	13.34285^{8}^
		$\hat{\eta }$	5.57580^{6}^	8.15722^{7}^	4.11647^{5}^	3.53950^{3}^	8.54848^{8}^	3.59909^{4}^	2.08577^{2}^	0.33228^{1}^
	MRE	$\hat{\alpha }$	0.20442^{3}^	0.19791^{2}^	0.25899^{7}^	0.20899^{5}^	0.21335^{6}^	0.19176^{1}^	0.20835^{4}^	0.29170^{8}^
		$\hat{\sigma }$	1.06332^{3}^	0.91389^{1}^	2.32372^{7}^	1.06712^{4}^	1.07845^{5}^	1.04043^{2}^	2.01529^{6}^	3.73285^{8}^
		$\hat{\eta }$	2.35450^{6}^	3.00974^{7}^	1.80746^{4}^	1.82520^{5}^	3.07124^{8}^	1.79356^{3}^	1.21267^{2}^	0.76093^{1}^
	∑ *Ranks*		37^{3}^	30^{2}^	55^{8}^	40^{5}^	53^{7}^	20^{1}^	38^{4}^	51^{6}^
80	|*BIAS*|	$\hat{\alpha }$	0.13999^{4}^	0.13506^{3}^	0.16769^{7}^	0.13276^{1}^	0.14036^{5}^	0.13357^{2}^	0.14174^{6}^	0.19642^{8}^
		$\hat{\sigma }$	0.47437^{5}^	0.40180^{1}^	0.88682^{7}^	0.42589^{2}^	0.44882^{4}^	0.43819^{3}^	0.84282^{6}^	1.38751^{8}^
		$\hat{\eta }$	0.54779^{6}^	0.79594^{8}^	0.45179^{4}^	0.45071^{3}^	0.73463^{7}^	0.48233^{5}^	0.32790^{2}^	0.27702^{1}^
	MSE	$\hat{\alpha }$	0.02939^{4}^	0.02803^{3}^	0.04338^{7}^	0.02686^{1}^	0.02981^{5}^	0.02760^{2}^	0.03543^{6}^	0.05339^{8}^
		$\hat{\sigma }$	0.80195^{4}^	0.46644^{1}^	3.15566^{6}^	0.87804^{5}^	0.60462^{2}^	0.63296^{3}^	3.48236^{7}^	8.66548^{8}^
		$\hat{\eta }$	1.98441^{6}^	4.33280^{8}^	1.57867^{4}^	1.39159^{3}^	3.89433^{7}^	1.58774^{5}^	0.81346^{2}^	0.11051^{1}^
	MRE	$\hat{\alpha }$	0.18665^{4}^	0.18008^{3}^	0.22358^{7}^	0.17702^{1}^	0.18715^{5}^	0.17810^{2}^	0.18899^{6}^	0.26189^{8}^
		$\hat{\sigma }$	0.94874^{5}^	0.80360^{1}^	1.77365^{7}^	0.85178^{2}^	0.89765^{4}^	0.87638^{3}^	1.68564^{6}^	2.77501^{8}^
		$\hat{\eta }$	1.36947^{6}^	1.98985^{8}^	1.12946^{4}^	1.12677^{3}^	1.83657^{7}^	1.20583^{5}^	0.81976^{2}^	0.69254^{1}^
	∑ *Ranks*		44^{5}^	36^{3}^	53^{8}^	21^{1}^	46^{6}^	30^{2}^	43^{4}^	51^{7}^
120	|*BIAS*|	$\hat{\alpha }$	0.12537^{3}^	0.12633^{5}^	0.14261^{7}^	0.11719^{1}^	0.12956^{6}^	0.12199^{2}^	0.12554^{4}^	0.18126^{8}^
		$\hat{\sigma }$	0.41757^{5}^	0.37998^{2}^	0.66589^{7}^	0.34457^{1}^	0.41147^{4}^	0.40049^{3}^	0.61187^{6}^	1.02316^{8}^
		$\hat{\eta }$	0.37192^{6}^	0.50326^{8}^	0.30319^{3}^	0.31866^{4}^	0.48670^{7}^	0.33973^{5}^	0.24388^{1}^	0.27195^{2}^
	MSE	$\hat{\alpha }$	0.02400^{3}^	0.02450^{4}^	0.03163^{7}^	0.02078^{1}^	0.02519^{5}^	0.02299^{2}^	0.02753^{6}^	0.04357^{8}^
		$\hat{\sigma }$	0.56223^{5}^	0.38907^{1}^	1.62499^{6}^	0.47378^{3}^	0.46165^{2}^	0.49343^{4}^	1.70282^{7}^	5.09889^{8}^
		$\hat{\eta }$	0.80403^{6}^	1.73816^{8}^	0.51922^{4}^	0.51761^{3}^	1.65565^{7}^	0.57520^{5}^	0.27452^{2}^	0.09841^{1}^
	MRE	$\hat{\alpha }$	0.16716^{3}^	0.16843^{5}^	0.19015^{7}^	0.15625^{1}^	0.17275^{6}^	0.16266^{2}^	0.16738^{4}^	0.24168^{8}^
		$\hat{\sigma }$	0.83514^{5}^	0.75995^{2}^	1.33178^{7}^	0.68915^{1}^	0.82295^{4}^	0.80097^{3}^	1.22375^{6}^	2.04632^{8}^
		$\hat{\eta }$	0.92979^{6}^	1.25815^{8}^	0.75797^{3}^	0.79665^{4}^	1.21674^{7}^	0.84932^{5}^	0.60971^{1}^	0.67987^{2}^
	∑ *Ranks*		42^{4}^	43^{5}^	51^{7}^	19^{1}^	48^{6}^	31^{2}^	37^{3}^	53^{8}^
200	|*BIAS*|	$\hat{\alpha }$	0.11124^{3}^	0.11204^{4}^	0.11904^{7}^	0.09764^{1}^	0.11526^{5}^	0.10660^{2}^	0.11598^{6}^	0.16527^{8}^
		$\hat{\sigma }$	0.35639^{5}^	0.32653^{2}^	0.47527^{6}^	0.25880^{1}^	0.35235^{4}^	0.33557^{3}^	0.49053^{7}^	0.72585^{8}^
		$\hat{\eta }$	0.23647^{5}^	0.31009^{7}^	0.21604^{2}^	0.21819^{3}^	0.31042^{8}^	0.23457^{4}^	0.19966^{1}^	0.27333^{6}^
	MSE	$\hat{\alpha }$	0.01862^{3}^	0.01913^{4}^	0.02207^{6}^	0.01474^{1}^	0.02004^{5}^	0.01764^{2}^	0.02241^{7}^	0.03503^{8}^
		$\hat{\sigma }$	0.33130^{5}^	0.25939^{2}^	0.71230^{6}^	0.21994^{1}^	0.30105^{4}^	0.29350^{3}^	0.84125^{7}^	2.53411^{8}^
		$\hat{\eta }$	0.16889^{4}^	0.47311^{7}^	0.18505^{6}^	0.12350^{3}^	0.52924^{8}^	0.17586^{5}^	0.10159^{2}^	0.10109^{1}^
	MRE	$\hat{\alpha }$	0.14832^{3}^	0.14939^{4}^	0.15873^{7}^	0.13018^{1}^	0.15368^{5}^	0.14213^{2}^	0.15463^{6}^	0.22036^{8}^
		$\hat{\sigma }$	0.71277^{5}^	0.65306^{2}^	0.95055^{6}^	0.51759^{1}^	0.70471^{4}^	0.67114^{3}^	0.98105^{7}^	1.45170^{8}^
		$\hat{\eta }$	0.59117^{5}^	0.77522^{7}^	0.54010^{2}^	0.54548^{3}^	0.77604^{8}^	0.58641^{4}^	0.49916^{1}^	0.68333^{6}^
	∑ *Ranks*		38^{3}^	39^{4}^	48^{6}^	15^{1}^	51^{7}^	28^{2}^	44^{5}^	61^{8}^
300	|*BIAS*|	$\hat{\alpha }$	0.09859^{3}^	0.10367^{5}^	0.10112^{4}^	0.08447^{1}^	0.10439^{6}^	0.09669^{2}^	0.10441^{7}^	0.15340^{8}^
		$\hat{\sigma }$	0.30224^{4}^	0.29735^{3}^	0.36353^{6}^	0.21459^{1}^	0.30838^{5}^	0.29675^{2}^	0.38897^{7}^	0.53587^{8}^
		$\hat{\eta }$	0.18787^{4}^	0.23870^{7}^	0.17273^{2}^	0.17190^{1}^	0.23597^{6}^	0.18974^{5}^	0.17422^{3}^	0.27968^{8}^
	MSE	$\hat{\alpha }$	0.01489^{3}^	0.01616^{5}^	0.01604^{4}^	0.01111^{1}^	0.01635^{6}^	0.01421^{2}^	0.01794^{7}^	0.02939^{8}^
		$\hat{\sigma }$	0.22391^{5}^	0.19251^{2}^	0.37750^{6}^	0.12612^{1}^	0.21515^{4}^	0.21307^{3}^	0.43664^{7}^	1.21509^{8}^
		$\hat{\eta }$	0.08185^{4}^	0.17627^{7}^	0.06773^{2}^	0.07077^{3}^	0.21979^{8}^	0.08577^{5}^	0.06617^{1}^	0.10929^{6}^
	MRE	$\hat{\alpha }$	0.13145^{3}^	0.13822^{5}^	0.13483^{4}^	0.11262^{1}^	0.13918^{6}^	0.12892^{2}^	0.13922^{7}^	0.20454^{8}^
		$\hat{\sigma }$	0.60448^{4}^	0.59470^{3}^	0.72706^{6}^	0.42918^{1}^	0.61676^{5}^	0.59350^{2}^	0.77794^{7}^	1.07175^{8}^
		$\hat{\eta }$	0.46968^{4}^	0.59676^{7}^	0.43181^{2}^	0.42975^{1}^	0.58992^{6}^	0.47436^{5}^	0.43554^{3}^	0.69920^{8}^
	∑ *Ranks*		34^{3}^	44^{5}^	36^{4}^	11^{1}^	52^{7}^	28^{2}^	49^{6}^	70^{8}^

**Table 4:** Simulation results for $\Theta ={\left(\eta =1.60,\sigma =1.75,\alpha =0.75\right)}^{⊺}$.

*n*	Est.	Est. Par.	WLSE	OLSE	MLE	MPSE	CVME	ADE	RADE	PCE
50	|*BIAS*|	$\hat{\alpha }$	0.15594^{4}^	0.16777^{5}^	0.15193^{3}^	0.13514^{1}^	0.16895^{6}^	0.15033^{2}^	0.17468^{7}^	0.20072^{8}^
		$\hat{\sigma }$	2.81177^{5}^	3.01535^{7}^	2.67854^{3}^	2.10315^{1}^	3.18103^{8}^	2.60251^{2}^	2.97845^{6}^	2.71654^{4}^
		$\hat{\eta }$	2.25005^{4}^	2.69460^{7}^	2.28043^{5}^	1.87179^{1}^	2.59819^{6}^	1.90736^{2}^	2.20983^{3}^	3.37086^{8}^
	MSE	$\hat{\alpha }$	0.03743^{4}^	0.04276^{5}^	0.03672^{3}^	0.02789^{1}^	0.04502^{6}^	0.03529^{2}^	0.04685^{7}^	0.05650^{8}^
		$\hat{\sigma }$	17.95112^{5}^	19.88132^{6}^	16.68591^{3}^	11.85546^{1}^	21.74625^{8}^	15.84872^{2}^	19.94607^{7}^	17.14619^{4}^
		$\hat{\eta }$	15.75786^{4}^	20.48024^{7}^	16.08911^{5}^	12.34565^{2}^	19.47114^{6}^	12.10946^{1}^	15.31724^{3}^	29.28544^{8}^
	MRE	$\hat{\alpha }$	0.20792^{4}^	0.22369^{5}^	0.20257^{3}^	0.18019^{1}^	0.22527^{6}^	0.20043^{2}^	0.23291^{7}^	0.26763^{8}^
		$\hat{\sigma }$	1.60673^{5}^	1.72306^{7}^	1.53059^{3}^	1.20180^{1}^	1.81773^{8}^	1.48715^{2}^	1.70197^{6}^	1.55231^{4}^
		$\hat{\eta }$	1.40628^{4}^	1.68412^{7}^	1.42527^{5}^	1.16987^{1}^	1.62387^{6}^	1.19210^{2}^	1.38114^{3}^	2.10679^{8}^
	∑ *Ranks*		39^{4}^	56^{6}^	33^{3}^	10^{1}^	60^{7.5}^	17^{2}^	49^{5}^	60^{7.5}^
80	|*BIAS*|	$\hat{\alpha }$	0.12497^{4}^	0.13517^{5}^	0.12459^{3}^	0.10820^{1}^	0.14012^{6}^	0.12350^{2}^	0.14858^{7}^	0.18716^{8}^
		$\hat{\sigma }$	2.13243^{4}^	2.43517^{5}^	2.11181^{3}^	1.58319^{1}^	2.49827^{8}^	2.08135^{2}^	2.47731^{7}^	2.44887^{6}^
		$\hat{\eta }$	1.60287^{4}^	2.11585^{7}^	1.48128^{3}^	1.16521^{1}^	2.03733^{6}^	1.36381^{2}^	1.63181^{5}^	2.83725^{8}^
	MSE	$\hat{\alpha }$	0.02480^{3}^	0.02830^{5}^	0.02503^{4}^	0.01873^{1}^	0.03144^{6}^	0.02451^{2}^	0.03414^{7}^	0.05007^{8}^
		$\hat{\sigma }$	11.25858^{3}^	14.15113^{5}^	11.40344^{4}^	7.47799^{1}^	14.65177^{7}^	10.86452^{2}^	14.41922^{6}^	15.29300^{8}^
		$\hat{\eta }$	9.38092^{4}^	14.62101^{7}^	8.27950^{3}^	5.86636^{1}^	13.81730^{6}^	7.04175^{2}^	9.64414^{5}^	23.92091^{8}^
	MRE	$\hat{\alpha }$	0.16663^{4}^	0.18023^{5}^	0.16612^{3}^	0.14426^{1}^	0.18683^{6}^	0.16467^{2}^	0.19810^{7}^	0.24955^{8}^
		$\hat{\sigma }$	1.21853^{4}^	1.39153^{5}^	1.20675^{3}^	0.90468^{1}^	1.42758^{8}^	1.18935^{2}^	1.41561^{7}^	1.39936^{6}^
		$\hat{\eta }$	1.00179^{4}^	1.32241^{7}^	0.92580^{3}^	0.72826^{1}^	1.27333^{6}^	0.85238^{2}^	1.01988^{5}^	1.77328^{8}^
	∑ *Ranks*		34^{4}^	51^{5}^	29^{3}^	9^{1}^	59^{7}^	18^{2}^	56^{6}^	68^{8}^
120	|*BIAS*|	$\hat{\alpha }$	0.10453^{4}^	0.11859^{5}^	0.10254^{2}^	0.08792^{1}^	0.12056^{6}^	0.10410^{3}^	0.12753^{7}^	0.18095^{8}^
		$\hat{\sigma }$	1.69639^{4}^	1.97554^{5}^	1.58936^{2}^	1.16395^{1}^	2.04628^{7}^	1.64233^{3}^	2.00605^{6}^	2.30887^{8}^
		$\hat{\eta }$	1.16239^{4}^	1.64755^{7}^	1.03752^{3}^	0.78374^{1}^	1.58413^{6}^	1.03642^{2}^	1.25049^{5}^	2.41371^{8}^
	MSE	$\hat{\alpha }$	0.01765^{3}^	0.02202^{5}^	0.01718^{2}^	0.01268^{1}^	0.02345^{6}^	0.01783^{4}^	0.02557^{7}^	0.04708^{8}^
		$\hat{\sigma }$	7.43074^{4}^	9.60697^{5}^	6.45201^{2}^	4.29625^{1}^	10.36250^{7}^	6.77098^{3}^	10.18328^{6}^	14.63117^{8}^
		$\hat{\eta }$	5.42686^{4}^	9.96393^{7}^	4.34960^{3}^	2.68689^{1}^	9.25849^{6}^	4.16922^{2}^	5.85774^{5}^	19.44803^{8}^
	MRE	$\hat{\alpha }$	0.13937^{4}^	0.15812^{5}^	0.13672^{2}^	0.11722^{1}^	0.16074^{6}^	0.13880^{3}^	0.17004^{7}^	0.24127^{8}^
		$\hat{\sigma }$	0.96937^{4}^	1.12888^{5}^	0.90821^{2}^	0.66512^{1}^	1.16930^{7}^	0.93847^{3}^	1.14631^{6}^	1.31936^{8}^
		$\hat{\eta }$	0.72649^{4}^	1.02972^{7}^	0.64845^{3}^	0.48984^{1}^	0.99008^{6}^	0.64776^{2}^	0.78156^{5}^	1.50857^{8}^
	∑ *Ranks*		35^{4}^	51^{5}^	21^{2}^	9^{1}^	57^{7}^	25^{3}^	54^{6}^	72^{8}^
200	|*BIAS*|	$\hat{\alpha }$	0.08098^{4}^	0.09298^{5}^	0.07713^{2}^	0.06555^{1}^	0.09510^{6}^	0.08079^{3}^	0.09758^{7}^	0.16932^{8}^
		$\hat{\sigma }$	1.17732^{3}^	1.47745^{7}^	1.11485^{2}^	0.77192^{1}^	1.46222^{6}^	1.18219^{4}^	1.42105^{5}^	2.12258^{8}^
		$\hat{\eta }$	0.75054^{4}^	1.06170^{6}^	0.61615^{2}^	0.48076^{1}^	1.08878^{7}^	0.66802^{3}^	0.79856^{5}^	1.83963^{8}^
	MSE	$\hat{\alpha }$	0.01066^{4}^	0.01378^{5}^	0.00982^{2}^	0.00734^{1}^	0.01459^{6}^	0.01057^{3}^	0.01563^{7}^	0.04360^{8}^
		$\hat{\sigma }$	3.30847^{3}^	5.24636^{6}^	3.02599^{2}^	1.99329^{1}^	5.28065^{7}^	3.31036^{4}^	5.18396^{5}^	13.65971^{8}^
		$\hat{\eta }$	2.12574^{4}^	4.66485^{6}^	1.28918^{2}^	0.96751^{1}^	4.68480^{7}^	1.49409^{3}^	2.36137^{5}^	13.88671^{8}^
	MRE	$\hat{\alpha }$	0.10797^{4}^	0.12397^{5}^	0.10283^{2}^	0.08740^{1}^	0.12679^{6}^	0.10772^{3}^	0.13011^{7}^	0.22576^{8}^
		$\hat{\sigma }$	0.67275^{3}^	0.84426^{7}^	0.63706^{2}^	0.44109^{1}^	0.83556^{6}^	0.67554^{4}^	0.81203^{5}^	1.21290^{8}^
		$\hat{\eta }$	0.46908^{4}^	0.66356^{6}^	0.38509^{2}^	0.30048^{1}^	0.68048^{7}^	0.41751^{3}^	0.49910^{5}^	1.14977^{8}^
	∑ *Ranks*		33^{4}^	53^{6}^	18^{2}^	9^{1}^	58^{7}^	30^{3}^	51^{5}^	72^{8}^
300	|*BIAS*|	$\hat{\alpha }$	0.06462^{4}^	0.07761^{6}^	0.06253^{2}^	0.05167^{1}^	0.07688^{5}^	0.06378^{3}^	0.08279^{7}^	0.16828^{8}^
		$\hat{\sigma }$	0.89709^{4}^	1.15175^{7}^	0.86701^{2}^	0.52358^{1}^	1.14385^{6}^	0.88772^{3}^	1.13767^{5}^	2.20658^{8}^
		$\hat{\eta }$	0.51012^{4}^	0.73450^{7}^	0.44203^{2}^	0.31173^{1}^	0.70870^{6}^	0.47766^{3}^	0.56995^{5}^	1.51816^{8}^
	MSE	$\hat{\alpha }$	0.00684^{4}^	0.00967^{6}^	0.00643^{2}^	0.00481^{1}^	0.00954^{5}^	0.00669^{3}^	0.01141^{7}^	0.04465^{8}^
		$\hat{\sigma }$	1.77045^{3}^	3.14406^{7}^	1.66765^{2}^	1.06840^{1}^	2.95991^{5}^	1.79587^{4}^	3.13051^{6}^	14.82925^{8}^
		$\hat{\eta }$	0.71236^{4}^	1.95459^{7}^	0.48771^{2}^	0.34169^{1}^	1.75975^{6}^	0.62947^{3}^	0.94202^{5}^	10.45907^{8}^
	MRE	$\hat{\alpha }$	0.08617^{4}^	0.10347^{6}^	0.08337^{2}^	0.06890^{1}^	0.10251^{5}^	0.08504^{3}^	0.11039^{7}^	0.22438^{8}^
		$\hat{\sigma }$	0.51262^{4}^	0.65814^{7}^	0.49543^{2}^	0.29919^{1}^	0.65363^{6}^	0.50727^{3}^	0.65010^{5}^	1.26091^{8}^
		$\hat{\eta }$	0.31882^{4}^	0.45906^{7}^	0.27627^{2}^	0.19483^{1}^	0.44294^{6}^	0.29854^{3}^	0.35622^{5}^	0.94885^{8}^
	∑ *Ranks*		35^{4}^	60^{7}^	18^{2}^	9^{1}^	50^{5}^	28^{3}^	52^{6}^	72^{8}^

**Table 5:** Simulation results for $\Theta ={\left(\eta =0.40,\sigma =0.50,\alpha =1.50\right)}^{⊺}$.

*n*	Est.	Est. Par.	WLSE	OLSE	MLE	MPSE	CVME	ADE	RADE	PCE
50	|*BIAS*|	$\hat{\alpha }$	0.30850^{6}^	0.30558^{4}^	0.38386^{8}^	0.30489^{3}^	0.31677^{7}^	0.28994^{2}^	0.28944^{1}^	0.30577^{5}^
		$\hat{\sigma }$	0.55417^{3}^	0.56060^{4}^	1.15678^{8}^	0.49726^{1}^	0.60143^{5}^	0.54397^{2}^	0.88206^{6}^	0.88913^{7}^
		$\hat{\eta }$	0.85251^{6}^	1.16302^{7}^	0.75175^{5}^	0.74878^{4}^	1.18855^{8}^	0.73018^{3}^	0.51561^{2}^	0.43315^{1}^
	MSE	$\hat{\alpha }$	0.14175^{5}^	0.14072^{4}^	0.22715^{8}^	0.13799^{2}^	0.14976^{7}^	0.13089^{1}^	0.14412^{6}^	0.14011^{3}^
		$\hat{\sigma }$	1.40435^{3}^	1.40436^{4}^	5.45627^{8}^	1.39001^{2}^	1.51025^{5}^	1.35228^{1}^	3.99057^{6}^	4.19673^{7}^
		$\hat{\eta }$	4.60954^{6}^	7.91390^{7}^	4.32001^{5}^	3.71710^{4}^	8.35529^{8}^	3.65593^{3}^	2.34274^{2}^	1.70930^{1}^
	MRE	$\hat{\alpha }$	0.20567^{6}^	0.20372^{4}^	0.25591^{8}^	0.20326^{3}^	0.21118^{7}^	0.19329^{2}^	0.19296^{1}^	0.20385^{5}^
		$\hat{\sigma }$	1.10834^{3}^	1.12120^{4}^	2.31357^{8}^	0.99452^{1}^	1.20286^{5}^	1.08793^{2}^	1.76413^{6}^	1.77826^{7}^
		$\hat{\eta }$	2.13128^{6}^	2.90754^{7}^	1.87938^{5}^	1.87195^{4}^	2.97137^{8}^	1.82546^{3}^	1.28902^{2}^	1.08289^{1}^
	∑ *Ranks*		44^{5}^	45^{6}^	63^{8}^	24^{2}^	60^{7}^	19^{1}^	32^{3}^	37^{4}^
80	|*BIAS*|	$\hat{\alpha }$	0.27248^{5}^	0.27089^{3}^	0.33498^{8}^	0.26785^{2}^	0.28358^{6}^	0.26776^{1}^	0.27094^{4}^	0.28429^{7}^
		$\hat{\sigma }$	0.46981^{4}^	0.43029^{2}^	0.87570^{8}^	0.42182^{1}^	0.48497^{5}^	0.44646^{3}^	0.76599^{7}^	0.72973^{6}^
		$\hat{\eta }$	0.53697^{6}^	0.76100^{8}^	0.46847^{4}^	0.45376^{3}^	0.75095^{7}^	0.50729^{5}^	0.36835^{2}^	0.28291^{1}^
	MSE	$\hat{\alpha }$	0.11152^{4}^	0.11146^{3}^	0.17114^{8}^	0.10721^{1}^	0.12087^{5}^	0.10892^{2}^	0.12715^{7}^	0.12330^{6}^
		$\hat{\sigma }$	0.82018^{3}^	0.66498^{1}^	3.08751^{8}^	0.86473^{5}^	0.85409^{4}^	0.67640^{2}^	2.88842^{7}^	2.83468^{6}^
		$\hat{\eta }$	1.95677^{6}^	4.06384^{8}^	1.76801^{5}^	1.28518^{3}^	4.05259^{7}^	1.66177^{4}^	1.04532^{2}^	0.53845^{1}^
	MRE	$\hat{\alpha }$	0.18166^{5}^	0.18059^{3}^	0.22332^{8}^	0.17857^{2}^	0.18906^{6}^	0.17851^{1}^	0.18063^{4}^	0.18953^{7}^
		$\hat{\sigma }$	0.93962^{4}^	0.86059^{2}^	1.75141^{8}^	0.84365^{1}^	0.96993^{5}^	0.89292^{3}^	1.53197^{7}^	1.45946^{6}^
		$\hat{\eta }$	1.34242^{6}^	1.90250^{8}^	1.17118^{4}^	1.13439^{3}^	1.87737^{7}^	1.26824^{5}^	0.92087^{2}^	0.70727^{1}^
	∑ *Ranks*		43^{6}^	38^{3}^	61^{8}^	21^{1}^	52^{7}^	26^{2}^	42^{5}^	41^{4}^
120	|*BIAS*|	$\hat{\alpha }$	0.25233^{5}^	0.25147^{4}^	0.28944^{8}^	0.23530^{1}^	0.25722^{7}^	0.24175^{2}^	0.24933^{3}^	0.25312^{6}^
		$\hat{\sigma }$	0.42373^{5}^	0.39937^{3}^	0.66114^{8}^	0.34242^{1}^	0.40730^{4}^	0.39921^{2}^	0.61200^{7}^	0.53078^{6}^
		$\hat{\eta }$	0.35552^{6}^	0.51764^{8}^	0.31304^{3}^	0.32325^{4}^	0.48150^{7}^	0.32548^{5}^	0.27485^{2}^	0.22298^{1}^
	MSE	$\hat{\alpha }$	0.09640^{3}^	0.09747^{5}^	0.12795^{8}^	0.08374^{1}^	0.09896^{6}^	0.08916^{2}^	0.10582^{7}^	0.09679^{4}^
		$\hat{\sigma }$	0.58138^{5}^	0.46634^{2}^	1.59519^{8}^	0.47202^{3}^	0.45436^{1}^	0.47387^{4}^	1.59063^{7}^	1.32239^{6}^
		$\hat{\eta }$	0.64953^{6}^	1.97967^{8}^	0.60286^{5}^	0.48917^{4}^	1.67427^{7}^	0.46123^{3}^	0.45773^{2}^	0.24647^{1}^
	MRE	$\hat{\alpha }$	0.16822^{5}^	0.16765^{4}^	0.19296^{8}^	0.15687^{1}^	0.17148^{7}^	0.16116^{2}^	0.16622^{3}^	0.16875^{6}^
		$\hat{\sigma }$	0.84747^{5}^	0.79875^{3}^	1.32229^{8}^	0.68485^{1}^	0.81460^{4}^	0.79842^{2}^	1.22399^{7}^	1.06156^{6}^
		$\hat{\eta }$	0.88880^{6}^	1.29410^{8}^	0.78259^{3}^	0.80812^{4}^	1.20376^{7}^	0.81369^{5}^	0.68712^{2}^	0.55745^{1}^
	∑ *Ranks*		46^{6}^	45^{5}^	59^{8}^	20^{1}^	50^{7}^	27^{2}^	40^{4}^	37^{3}^
200	|*BIAS*|	$\hat{\alpha }$	0.21877^{3}^	0.21878^{4}^	0.23386^{8}^	0.19352^{1}^	0.23240^{7}^	0.21100^{2}^	0.22417^{5}^	0.22907^{6}^
		$\hat{\sigma }$	0.34688^{4}^	0.33084^{2}^	0.46123^{7}^	0.26088^{1}^	0.35587^{5}^	0.33636^{3}^	0.46140^{8}^	0.42022^{6}^
		$\hat{\eta }$	0.23049^{6}^	0.30450^{7}^	0.21805^{3}^	0.21981^{4}^	0.30909^{8}^	0.22967^{5}^	0.20512^{2}^	0.18015^{1}^
	MSE	$\hat{\alpha }$	0.07206^{3}^	0.07341^{4}^	0.08553^{8}^	0.05892^{1}^	0.08068^{6}^	0.06947^{2}^	0.08417^{7}^	0.07662^{5}^
		$\hat{\sigma }$	0.32709^{5}^	0.27729^{2}^	0.71354^{7}^	0.21643^{1}^	0.31236^{4}^	0.30524^{3}^	0.72814^{8}^	0.68365^{6}^
		$\hat{\eta }$	0.14735^{4}^	0.51264^{7}^	0.18591^{6}^	0.14099^{3}^	0.52009^{8}^	0.14756^{5}^	0.11550^{2}^	0.05895^{1}^
	MRE	$\hat{\alpha }$	0.14585^{3}^	0.14586^{4}^	0.15591^{8}^	0.12902^{1}^	0.15494^{7}^	0.14067^{2}^	0.14944^{5}^	0.15272^{6}^
		$\hat{\sigma }$	0.69377^{4}^	0.66168^{2}^	0.92246^{7}^	0.52176^{1}^	0.71173^{5}^	0.67272^{3}^	0.92281^{8}^	0.84044^{6}^
		$\hat{\eta }$	0.57622^{6}^	0.76124^{7}^	0.54512^{3}^	0.54952^{4}^	0.77271^{8}^	0.57418^{5}^	0.51281^{2}^	0.45037^{1}^
	∑ *Ranks*		38^{3.5}^	39^{5}^	57^{7}^	17^{1}^	58^{8}^	30^{2}^	47^{6}^	38^{3.5}^
300	|*BIAS*|	$\hat{\alpha }$	0.19901^{3}^	0.20452^{6}^	0.20135^{4}^	0.16332^{1}^	0.21069^{7}^	0.18630^{2}^	0.20248^{5}^	0.21379^{8}^
		$\hat{\sigma }$	0.31159^{3}^	0.31190^{4}^	0.36501^{7}^	0.21128^{1}^	0.31294^{5}^	0.27780^{2}^	0.37733^{8}^	0.36180^{6}^
		$\hat{\eta }$	0.18766^{6}^	0.21310^{7}^	0.17260^{3}^	0.16852^{1}^	0.22783^{8}^	0.18643^{5}^	0.17427^{4}^	0.16905^{2}^
	MSE	$\hat{\alpha }$	0.06006^{3}^	0.06354^{5}^	0.06277^{4}^	0.04359^{1}^	0.06660^{7}^	0.05349^{2}^	0.06780^{8}^	0.06626^{6}^
		$\hat{\sigma }$	0.24224^{5}^	0.22910^{4}^	0.37515^{6}^	0.11738^{1}^	0.22154^{3}^	0.18934^{2}^	0.42823^{8}^	0.42194^{7}^
		$\hat{\eta }$	0.07768^{5}^	0.11457^{7}^	0.06897^{3}^	0.06931^{4}^	0.17962^{8}^	0.08367^{6}^	0.06895^{2}^	0.04770^{1}^
	MRE	$\hat{\alpha }$	0.13267^{3}^	0.13635^{6}^	0.13424^{4}^	0.10888^{1}^	0.14046^{7}^	0.12420^{2}^	0.13498^{5}^	0.14253^{8}^
		$\hat{\sigma }$	0.62318^{3}^	0.62380^{4}^	0.73002^{7}^	0.42256^{1}^	0.62587^{5}^	0.55561^{2}^	0.75467^{8}^	0.72361^{6}^
		$\hat{\eta }$	0.46914^{6}^	0.53274^{7}^	0.43150^{3}^	0.42129^{1}^	0.56957^{8}^	0.46607^{5}^	0.43568^{4}^	0.42263^{2}^
	∑ *Ranks*		37^{3}^	50^{6}^	41^{4}^	12^{1}^	58^{8}^	28^{2}^	52^{7}^	46^{5}^

**Table 6:** Simulation results for $\Theta ={\left(\eta =0.40,\sigma =3.00,\alpha =1.50\right)}^{⊺}$.

*n*	Est.	Est. Par.	WLSE	OLSE	MLE	MPSE	CVME	ADE	RADE	PCE
50	|*BIAS*|	$\hat{\alpha }$	0.33043^{6}^	0.32951^{5}^	0.31806^{3}^	0.29196^{1}^	0.33641^{7}^	0.30724^{2}^	0.32583^{4}^	0.37630^{8}^
		$\hat{\sigma }$	3.53120^{3}^	3.58269^{4}^	3.95734^{8}^	2.05586^{1}^	3.87746^{6}^	3.21566^{2}^	3.76597^{5}^	3.94080^{7}^
		$\hat{\eta }$	0.59201^{6}^	0.70283^{8}^	0.49736^{3}^	0.51330^{4}^	0.59701^{7}^	0.58083^{5}^	0.28698^{2}^	0.28384^{1}^
	MSE	$\hat{\alpha }$	0.15954^{5}^	0.16293^{6}^	0.14835^{3}^	0.13287^{1}^	0.17164^{7}^	0.14297^{2}^	0.15048^{4}^	0.18457^{8}^
		$\hat{\sigma }$	23.26186^{3}^	24.01224^{4}^	28.30320^{8}^	11.57715^{1}^	27.73259^{7}^	19.80058^{2}^	26.23122^{5}^	27.06776^{6}^
		$\hat{\eta }$	2.70685^{6}^	3.88216^{8}^	2.60330^{4}^	2.11201^{3}^	3.32278^{7}^	2.67709^{5}^	0.38868^{2}^	0.15713^{1}^
	MRE	$\hat{\alpha }$	0.22028^{6}^	0.21967^{5}^	0.21204^{3}^	0.19464^{1}^	0.22427^{7}^	0.20483^{2}^	0.21722^{4}^	0.25086^{8}^
		$\hat{\sigma }$	1.17707^{3}^	1.19423^{4}^	1.31911^{8}^	0.68529^{1}^	1.29249^{6}^	1.07189^{2}^	1.25532^{5}^	1.31360^{7}^
		$\hat{\eta }$	1.48002^{6}^	1.75706^{8}^	1.24341^{3}^	1.28324^{4}^	1.49253^{7}^	1.45207^{5}^	0.71746^{2}^	0.70961^{1}^
	∑ *Ranks*		44^{5}^	52^{7}^	43^{4}^	17^{1}^	61^{8}^	27^{2}^	33^{3}^	47^{6}^
80	|*BIAS*|	$\hat{\alpha }$	0.28926^{4}^	0.29824^{6}^	0.27111^{2}^	0.24061^{1}^	0.30612^{7}^	0.27308^{3}^	0.29622^{5}^	0.34747^{8}^
		$\hat{\sigma }$	3.20381^{3}^	3.22791^{4}^	3.35546^{6}^	1.68997^{1}^	3.50447^{8}^	2.92617^{2}^	3.40921^{7}^	3.32716^{5}^
		$\hat{\eta }$	0.35901^{5}^	0.47404^{8}^	0.31643^{4}^	0.30301^{3}^	0.41762^{7}^	0.37046^{6}^	0.23802^{1}^	0.28327^{2}^
	MSE	$\hat{\alpha }$	0.12338^{4}^	0.13482^{6}^	0.11000^{2}^	0.09566^{1}^	0.13932^{7}^	0.11275^{3}^	0.12411^{5}^	0.15909^{8}^
		$\hat{\sigma }$	20.02490^{3}^	20.48774^{5}^	21.68009^{6}^	9.16632^{1}^	23.66964^{8}^	16.98962^{2}^	22.12180^{7}^	20.15692^{4}^
		$\hat{\eta }$	0.95398^{4}^	1.94256^{8}^	1.01536^{5}^	0.70574^{3}^	1.59777^{7}^	1.06188^{6}^	0.17996^{2}^	0.14946^{1}^
	MRE	$\hat{\alpha }$	0.19284^{4}^	0.19883^{6}^	0.18074^{2}^	0.16041^{1}^	0.20408^{7}^	0.18206^{3}^	0.19748^{5}^	0.23164^{8}^
		$\hat{\sigma }$	1.06794^{3}^	1.07597^{4}^	1.11849^{6}^	0.56332^{1}^	1.16816^{8}^	0.97539^{2}^	1.13640^{7}^	1.10905^{5}^
		$\hat{\eta }$	0.89752^{5}^	1.18509^{8}^	0.79108^{4}^	0.75752^{3}^	1.04405^{7}^	0.92615^{6}^	0.59505^{1}^	0.70817^{2}^
	∑ *Ranks*		35^{3}^	55^{7}^	37^{4}^	15^{1}^	66^{8}^	33^{2}^	40^{5}^	43^{6}^
120	|*BIAS*|	$\hat{\alpha }$	0.25773^{4}^	0.28000^{6}^	0.22850^{2}^	0.18835^{1}^	0.28545^{7}^	0.24260^{3}^	0.26140^{5}^	0.31924^{8}^
		$\hat{\sigma }$	2.87597^{5}^	3.05464^{7}^	2.74298^{3}^	1.10204^{1}^	3.19463^{8}^	2.57488^{2}^	2.91084^{6}^	2.84975^{4}^
		$\hat{\eta }$	0.24010^{4}^	0.32776^{8}^	0.20058^{2}^	0.18462^{1}^	0.31091^{7}^	0.25673^{5}^	0.20717^{3}^	0.27936^{6}^
	MSE	$\hat{\alpha }$	0.09881^{5}^	0.11866^{6}^	0.07961^{2}^	0.06372^{1}^	0.12235^{7}^	0.09136^{3}^	0.09799^{4}^	0.13709^{8}^
		$\hat{\sigma }$	16.79175^{5}^	18.61763^{7}^	15.21522^{4}^	4.79986^{1}^	20.23190^{8}^	13.82589^{2}^	16.80429^{6}^	14.96920^{3}^
		$\hat{\eta }$	0.25137^{4}^	0.78203^{8}^	0.25753^{5}^	0.21226^{3}^	0.77783^{7}^	0.40031^{6}^	0.11561^{1}^	0.15134^{2}^
	MRE	$\hat{\alpha }$	0.17182^{4}^	0.18667^{6}^	0.15233^{2}^	0.12557^{1}^	0.19030^{7}^	0.16173^{3}^	0.17426^{5}^	0.21282^{8}^
		$\hat{\sigma }$	0.95866^{5}^	1.01821^{7}^	0.91433^{3}^	0.36735^{1}^	1.06488^{8}^	0.85829^{2}^	0.97028^{6}^	0.94992^{4}^
		$\hat{\eta }$	0.60025^{4}^	0.81940^{8}^	0.50145^{2}^	0.46155^{1}^	0.77727^{7}^	0.64182^{5}^	0.51792^{3}^	0.69840^{6}^
	∑ *Ranks*		40^{5}^	63^{7}^	25^{2}^	11^{1}^	66^{8}^	31^{3}^	39^{4}^	49^{6}^
200	|*BIAS*|	$\hat{\alpha }$	0.21531^{4}^	0.24063^{6}^	0.18487^{2}^	0.12937^{1}^	0.24364^{7}^	0.20355^{3}^	0.22240^{5}^	0.28775^{8}^
		$\hat{\sigma }$	2.35057^{4}^	2.55335^{7}^	2.14148^{3}^	0.67823^{1}^	2.75084^{8}^	2.12491^{2}^	2.36389^{5}^	2.40611^{6}^
		$\hat{\eta }$	0.16767^{4}^	0.21435^{7}^	0.13598^{2}^	0.10340^{1}^	0.19722^{6}^	0.17433^{5}^	0.16147^{3}^	0.25937^{8}^
	MSE	$\hat{\alpha }$	0.07103^{4}^	0.09087^{6}^	0.05387^{2}^	0.03533^{1}^	0.09158^{7}^	0.06713^{3}^	0.07292^{5}^	0.11636^{8}^
		$\hat{\sigma }$	11.51880^{6}^	13.78026^{7}^	9.71845^{3}^	2.85096^{1}^	15.70660^{8}^	9.61148^{2}^	11.28410^{5}^	10.45522^{4}^
		$\hat{\eta }$	0.07708^{4}^	0.20984^{8}^	0.06696^{3}^	0.05514^{1}^	0.17860^{7}^	0.10280^{5}^	0.06485^{2}^	0.14025^{6}^
	MRE	$\hat{\alpha }$	0.14354^{4}^	0.16042^{6}^	0.12325^{2}^	0.08625^{1}^	0.16243^{7}^	0.13570^{3}^	0.14826^{5}^	0.19183^{8}^
		$\hat{\sigma }$	0.78352^{4}^	0.85112^{7}^	0.71383^{3}^	0.22608^{1}^	0.91695^{8}^	0.70830^{2}^	0.78796^{5}^	0.80204^{6}^
		$\hat{\eta }$	0.41917^{4}^	0.53589^{7}^	0.33995^{2}^	0.25849^{1}^	0.49306^{6}^	0.43582^{5}^	0.40368^{3}^	0.64844^{8}^
	∑ *Ranks*		38^{4.5}^	61^{6}^	22^{2}^	9^{1}^	64^{8}^	30^{3}^	38^{4.5}^	62^{7}^
300	|*BIAS*|	$\hat{\alpha }$	0.17820^{4}^	0.21106^{6}^	0.15127^{2}^	0.09085^{1}^	0.21489^{7}^	0.16909^{3}^	0.19742^{5}^	0.25438^{8}^
		$\hat{\sigma }$	1.89922^{4}^	2.17749^{7}^	1.70010^{2}^	0.39175^{1}^	2.28127^{8}^	1.74144^{3}^	1.98304^{5}^	2.02589^{6}^
		$\hat{\eta }$	0.12475^{3}^	0.15834^{7}^	0.10134^{2}^	0.06372^{1}^	0.15613^{6}^	0.12651^{4}^	0.14412^{5}^	0.23857^{8}^
	MSE	$\hat{\alpha }$	0.05099^{4}^	0.07032^{6}^	0.03635^{2}^	0.01999^{1}^	0.07277^{7}^	0.04684^{3}^	0.05898^{5}^	0.09757^{8}^
		$\hat{\sigma }$	7.56035^{5}^	10.17147^{7}^	6.04658^{2}^	1.37027^{1}^	11.16689^{8}^	6.53991^{3}^	7.85512^{6}^	7.12519^{4}^
		$\hat{\eta }$	0.03781^{3}^	0.06757^{6}^	0.02627^{2}^	0.01982^{1}^	0.07444^{7}^	0.05211^{4}^	0.05247^{5}^	0.12845^{8}^
	MRE	$\hat{\alpha }$	0.11880^{4}^	0.14070^{6}^	0.10085^{2}^	0.06056^{1}^	0.14326^{7}^	0.11273^{3}^	0.13161^{5}^	0.16959^{8}^
		$\hat{\sigma }$	0.63307^{4}^	0.72583^{7}^	0.56670^{2}^	0.13058^{1}^	0.76042^{8}^	0.58048^{3}^	0.66101^{5}^	0.67530^{6}^
		$\hat{\eta }$	0.31187^{3}^	0.39585^{7}^	0.25334^{2}^	0.15929^{1}^	0.39032^{6}^	0.31628^{4}^	0.36029^{5}^	0.59644^{8}^
	∑ *Ranks*		34^{4}^	59^{6}^	18^{2}^	9^{1}^	64^{7.5}^	30^{3}^	46^{5}^	64^{7.5}^

**Table 7:** Simulation results for $\Theta ={\left(\eta =2.75,\sigma =3.00,\alpha =4.00\right)}^{⊺}$.

*n*	Est.	Est. Par.	WLSE	OLSE	MLE	MPSE	CVME	ADE	RADE	PCE
50	|*BIAS*|	$\hat{\alpha }$	0.59778^{5}^	0.62305^{6}^	0.56909^{3}^	0.54080^{1}^	0.64747^{7}^	0.59084^{4}^	0.66712^{8}^	0.56666^{2}^
		$\hat{\sigma }$	4.01899^{5}^	4.37494^{7}^	3.62756^{2}^	2.98797^{1}^	4.54045^{8}^	4.00520^{4}^	4.33139^{6}^	3.79293^{3}^
		$\hat{\eta }$	3.44613^{5}^	3.86638^{8}^	3.65043^{6}^	2.58380^{1}^	3.79483^{7}^	3.17264^{4}^	3.12316^{3}^	3.04529^{2}^
	MSE	$\hat{\alpha }$	0.52927^{5}^	0.58026^{6}^	0.48882^{3}^	0.42838^{1}^	0.62606^{7}^	0.52542^{4}^	0.65742^{8}^	0.46814^{2}^
		$\hat{\sigma }$	27.71628^{4}^	31.58603^{6}^	23.41150^{2}^	21.49446^{1}^	33.80749^{8}^	28.12566^{5}^	31.68135^{7}^	25.56214^{3}^
		$\hat{\eta }$	26.26666^{5}^	30.76079^{8}^	28.65321^{6}^	18.99484^{1}^	30.19341^{7}^	23.26666^{4}^	23.03320^{3}^	22.12216^{2}^
	MRE	$\hat{\alpha }$	0.14945^{5}^	0.15576^{6}^	0.14227^{3}^	0.13520^{1}^	0.16187^{7}^	0.14771^{4}^	0.16678^{8}^	0.14166^{2}^
		$\hat{\sigma }$	1.33966^{5}^	1.45831^{7}^	1.20919^{2}^	0.99599^{1}^	1.51348^{8}^	1.33507^{4}^	1.44380^{6}^	1.26431^{3}^
		$\hat{\eta }$	1.25314^{5}^	1.40596^{8}^	1.32743^{6}^	0.93956^{1}^	1.37994^{7}^	1.15369^{4}^	1.13569^{3}^	1.10738^{2}^
	∑ *Ranks*		44^{5}^	62^{7}^	33^{3}^	9^{1}^	66^{8}^	37^{4}^	52^{6}^	21^{2}^
80	|*BIAS*|	$\hat{\alpha }$	0.50617^{5}^	0.53885^{6}^	0.47970^{3}^	0.44161^{1}^	0.55987^{7}^	0.49723^{4}^	0.56928^{8}^	0.47848^{2}^
		$\hat{\sigma }$	3.52422^{5}^	3.80537^{7}^	3.04609^{2}^	2.31783^{1}^	4.02071^{8}^	3.32032^{4}^	3.72110^{6}^	3.22041^{3}^
		$\hat{\eta }$	2.80755^{5}^	3.47595^{8}^	2.94227^{6}^	1.94161^{1}^	3.41797^{7}^	2.65061^{4}^	2.62245^{3}^	2.38901^{2}^
	MSE	$\hat{\alpha }$	0.38057^{5}^	0.42601^{6}^	0.35254^{3}^	0.30419^{1}^	0.46329^{7}^	0.37710^{4}^	0.48387^{8}^	0.33793^{2}^
		$\hat{\sigma }$	22.57525^{5}^	25.60820^{7}^	17.47265^{2}^	15.82308^{1}^	27.89190^{8}^	20.42894^{4}^	25.06414^{6}^	19.60321^{3}^
		$\hat{\eta }$	19.74265^{5}^	26.72145^{8}^	21.09913^{6}^	13.09846^{1}^	26.01836^{7}^	17.93688^{4}^	17.54241^{3}^	15.35090^{2}^
	MRE	$\hat{\alpha }$	0.12654^{5}^	0.13471^{6}^	0.11992^{3}^	0.11040^{1}^	0.13997^{7}^	0.12431^{4}^	0.14232^{8}^	0.11962^{2}^
		$\hat{\sigma }$	1.17474^{5}^	1.26846^{7}^	1.01536^{2}^	0.77261^{1}^	1.34024^{8}^	1.10677^{4}^	1.24037^{6}^	1.07347^{3}^
		$\hat{\eta }$	1.02093^{5}^	1.26398^{8}^	1.06992^{6}^	0.70604^{1}^	1.24290^{7}^	0.96386^{4}^	0.95362^{3}^	0.86873^{2}^
	∑ *Ranks*		45^{5}^	63^{7}^	33^{3}^	9^{1}^	66^{8}^	36^{4}^	51^{6}^	21^{2}^
120	|*BIAS*|	$\hat{\alpha }$	0.42363^{4}^	0.47804^{6}^	0.41398^{3}^	0.35922^{1}^	0.48324^{7}^	0.43781^{5}^	0.49808^{8}^	0.40496^{2}^
		$\hat{\sigma }$	2.84404^{4}^	3.49487^{7}^	2.56244^{2}^	1.73307^{1}^	3.54903^{8}^	2.89614^{5}^	3.18526^{6}^	2.65429^{3}^
		$\hat{\eta }$	2.32527^{6}^	3.07348^{8}^	2.21402^{3}^	1.33401^{1}^	3.03364^{7}^	2.27492^{5}^	2.22162^{4}^	1.80094^{2}^
	MSE	$\hat{\alpha }$	0.27612^{4}^	0.34124^{6}^	0.26120^{3}^	0.20999^{1}^	0.34990^{7}^	0.28877^{5}^	0.37269^{8}^	0.24695^{2}^
		$\hat{\sigma }$	15.60259^{4}^	22.18507^{7}^	12.83558^{2}^	11.02411^{1}^	22.70225^{8}^	16.17781^{5}^	19.27970^{6}^	13.98475^{3}^
		$\hat{\eta }$	14.78504^{6}^	22.25792^{8}^	13.98403^{5}^	7.76497^{1}^	22.01203^{7}^	13.93878^{4}^	13.40289^{3}^	9.76685^{2}^
	MRE	$\hat{\alpha }$	0.10591^{4}^	0.11951^{6}^	0.10349^{3}^	0.08981^{1}^	0.12081^{7}^	0.10945^{5}^	0.12452^{8}^	0.10124^{2}^
		$\hat{\sigma }$	0.94801^{4}^	1.16496^{7}^	0.85415^{2}^	0.57769^{1}^	1.18301^{8}^	0.96538^{5}^	1.06175^{6}^	0.88476^{3}^
		$\hat{\eta }$	0.84555^{6}^	1.11763^{8}^	0.80510^{3}^	0.48509^{1}^	1.10314^{7}^	0.82724^{5}^	0.80786^{4}^	0.65489^{2}^
	∑ *Ranks*		42^{4}^	63^{7}^	26^{3}^	9^{1}^	66^{8}^	44^{5}^	53^{6}^	21^{2}^
200	|*BIAS*|	$\hat{\alpha }$	0.36106^{5}^	0.39648^{6}^	0.33331^{3}^	0.26169^{1}^	0.40080^{8}^	0.35807^{4}^	0.39808^{7}^	0.32750^{2}^
		$\hat{\sigma }$	2.39329^{5}^	2.84371^{7}^	2.00436^{2}^	1.05600^{1}^	2.89443^{8}^	2.32114^{4}^	2.43874^{6}^	2.06771^{3}^
		$\hat{\eta }$	1.76060^{6}^	2.34049^{7}^	1.54928^{3}^	0.78289^{1}^	2.39233^{8}^	1.63422^{4}^	1.64829^{5}^	1.31134^{2}^
	MSE	$\hat{\alpha }$	0.19922^{5}^	0.23514^{6}^	0.17644^{3}^	0.12232^{1}^	0.24493^{7}^	0.19855^{4}^	0.24606^{8}^	0.16617^{2}^
		$\hat{\sigma }$	11.44793^{5}^	15.47287^{7}^	8.10331^{2}^	6.02988^{1}^	15.96482^{8}^	10.85564^{4}^	12.12454^{6}^	8.80361^{3}^
		$\hat{\eta }$	9.32271^{6}^	14.71333^{7}^	7.30072^{3}^	3.61397^{1}^	15.52197^{8}^	7.88701^{4}^	8.11954^{5}^	5.40702^{2}^
	MRE	$\hat{\alpha }$	0.09027^{5}^	0.09912^{6}^	0.08333^{3}^	0.06542^{1}^	0.10020^{8}^	0.08952^{4}^	0.09952^{7}^	0.08188^{2}^
		$\hat{\sigma }$	0.79776^{5}^	0.94790^{7}^	0.66812^{2}^	0.35200^{1}^	0.96481^{8}^	0.77371^{4}^	0.81291^{6}^	0.68924^{3}^
		$\hat{\eta }$	0.64022^{6}^	0.85109^{7}^	0.56337^{3}^	0.28469^{1}^	0.86994^{8}^	0.59426^{4}^	0.59938^{5}^	0.47685^{2}^
	∑ *Ranks*		48^{5}^	60^{7}^	24^{3}^	9^{1}^	71^{8}^	36^{4}^	55^{6}^	21^{2}^
300	|*BIAS*|	$\hat{\alpha }$	0.29371^{5}^	0.34103^{7}^	0.26663^{2}^	0.18757^{1}^	0.33573^{6}^	0.28885^{4}^	0.34306^{8}^	0.27215^{3}^
		$\hat{\sigma }$	1.90248^{5}^	2.36207^{7}^	1.55036^{2}^	0.59564^{1}^	2.38574^{8}^	1.83038^{4}^	2.00820^{6}^	1.70138^{3}^
		$\hat{\eta }$	1.28116^{5}^	1.92314^{7}^	1.09330^{3}^	0.40539^{1}^	1.93477^{8}^	1.20322^{4}^	1.30225^{6}^	0.95929^{2}^
	MSE	$\hat{\alpha }$	0.13783^{5}^	0.17828^{7}^	0.11291^{2}^	0.06696^{1}^	0.17561^{6}^	0.13178^{4}^	0.18387^{8}^	0.11782^{3}^
		$\hat{\sigma }$	7.32500^{5}^	11.04248^{7}^	4.65699^{2}^	3.01634^{1}^	11.25531^{8}^	6.66935^{4}^	8.06946^{6}^	5.79055^{3}^
		$\hat{\eta }$	5.11281^{5}^	10.81770^{8}^	3.64730^{3}^	1.17628^{1}^	10.81108^{7}^	4.27450^{4}^	5.22644^{6}^	2.66789^{2}^
	MRE	$\hat{\alpha }$	0.07343^{5}^	0.08526^{7}^	0.06666^{2}^	0.04689^{1}^	0.08393^{6}^	0.07221^{4}^	0.08577^{8}^	0.06804^{3}^
		$\hat{\sigma }$	0.63416^{5}^	0.78736^{7}^	0.51679^{2}^	0.19855^{1}^	0.79525^{8}^	0.61013^{4}^	0.66940^{6}^	0.56713^{3}^
		$\hat{\eta }$	0.46587^{5}^	0.69932^{7}^	0.39757^{3}^	0.14741^{1}^	0.70355^{8}^	0.43754^{4}^	0.47354^{6}^	0.34883^{2}^
	∑ *Ranks*		45^{5}^	64^{7}^	21^{2}^	9^{1}^	65^{8}^	36^{4}^	60^{6}^	24^{3}^

In addition, Tables 3–7 show the rank of each estimator among all the remaining estimators in each row, with partial sum of the ranks for each column (∑ *Ranks*) for each sample size.

The partial and overall ranks of all estimators are shown in Table 8. From Table 8, we can conclude that the MPSE outperforms all the other estimators with an overall score of 142. Thus, for the NEHTW distribution we recommend the MPSE estimation method.

**Table 8:** Partial and overall ranks of all estimation methods for various combinations of Θ.

$\Theta^{⊺}$	*n*	WLSE	OLSE	MLE	MPSE	CVME	ADE	RADE	PCE
(*η* = 0.40, *σ* = 0.50, *α* = 0.75)	50	3	2	8	5	7	1	4	6
	80	5	3	8	1	6	2	4	7
	120	4	5	7	1	6	2	3	8
	200	3	4	6	1	7	2	5	8
	300	3	5	4	1	7	2	6	8
(*η* = 1.60, *σ* = 0.50, *α* = 0.75)	50	5	7	6	1	8	2	4	3
	80	3.5	7	5	1	8	2	6	3.5
	120	4	7	5	1	8	2	6	3
	200	3.5	6	3.5	1	7	2	8	5
	300	4	6.5	3	1	5	2	8	6.5
(*η* = 2.75, *σ* = 0.50, *α* = 0.75)	50	3	7	4	1	8	2	6	5
	80	4	6	3	1	8	2	5	7
	120	4	6	2	1	7	3	5	8
	200	4	7	2	1	6	3	5	8
	300	4	6	2	1	7	3	5	8
(*η* = 0.40, *σ* = 1.75, *α* = 0.75)	50	3.5	6	5	1	8	3.5	2	7
	80	5	7	2	1	8	3	4	6
	120	5	6	2.5	1	7	4	2.5	8
	200	4.5	6	2	1	7	3	4.5	8
	300	4	6	2	1	7	3	5	8
(*η* = 1.60, *σ* = 1.75, *α* = 0.75)	50	4	6	3	1	7.5	2	5	7.5
	80	4	5	3	1	7	2	6	8
	120	4	5	2	1	7	3	6	8
	200	4	6	2	1	7	3	5	8
	300	4	7	2	1	5	3	6	8
(*η* = 2.75, *σ* = 1.75, *α* = 0.75)	50	4	6	3	1	7	2	5	8
	80	4	6	3	1	7	2	5	8
	120	4	6	2	1	7	3	5	8
	200	4	6	2	1	7	3	5	8
	300	4	7	2	1	6	3	5	8
(*η* = 0.40, *σ* = 3.00, *α* = 0.75)	50	5	6	4	1	8	2	3	7
	80	4	6	5	1	8	3	2	7
	120	5	6	2	1	7	3	4	8
	200	4	6	2	1	7	3	5	8
	300	4	6	2	1	7	3	5	8
(*η* = 1.60, *σ* = 3.00, *α* = 0.75)	50	4	5	3	1	7	2	6	8
	80	4	5	2	1	7	3	6	8
	120	4	5.5	2	1	7	3	5.5	8
	200	4	7	2	1	6	3	5	8
	300	3	6	2	1	7	4	5	8
(*η* = 2.75, *σ* = 3.00, *α* = 0.75)	50	3.5	5	2	1	7	3.5	6	8
	80	4	6	3	1	7	2	5	8
	120	4	6	2.5	1	7	2.5	5	8
	200	4	7	2	1	6	3	5	8
	300	4	7	2	1	6	3	5	8
(*η* = 0.40, *σ* = 0.50, *α* = 1.50)	50	5	6	8	2	7	1	3	4
	80	6	3	8	1	7	2	5	4
	120	6	5	8	1	7	2	4	3
	200	3.5	5	7	1	8	2	6	3.5
	300	3	6	4	1	8	2	7	5
(*η* = 1.60, *σ* = 0.50, *α* = 1.50)	50	5	7	6	1	8	3	4	2
	80	4	7	6	1	8	3	5	2
	120	4	7	5	1	8	3	6	2
	200	5	6	4	1	7	2	8	3
	300	4	6	2	1	7	3	8	5
(*η* = 2.75, *σ* = 0.50, *α* = 1.50)	50	5	7	4	1	8	2	6	3
	80	3	7	4.5	1	8	2	6	4.5
	120	5	6	2.5	1	8	2.5	7	4
	200	4.5	7	2	1	8	3	6	4.5
	300	5	8	2	1	6.5	3	6.5	4
(*η* = 0.40, *σ* = 1.75, *α* = 1.50)	50	4	6	8	2	7	1	3	5
	80	6	7	5	1	8	2.5	2.5	4
	120	6	7	2	1	8	3	4	5
	200	4.5	8	2	1	7	3	4.5	6
	300	4	8	2	1	7	3	5	6
(*η* = 1.60, *σ* = 1.75, *α* = 1.50)	50	3	6	4	1	8	2	7	5
	80	4	7	3	1	8	2	6	5
	120	4	5	2	1	8	3	7	6
	200	4	7.5	2	1	5	3	6	7.5
	300	3	6.5	2	1	6.5	4	5	8
(*η* = 2.75, *σ* = 1.75, *α* = 1.50)	50	3	6	2	1	8	4	7	5
	80	4	7	3	1	8	2	5	6
	120	4	7	2.5	1	8	2.5	5	6
	200	4	7	2	1	8	3	5	6
	300	4	8	2	1	6	3	5	7
(*η* = 0.40, *σ* = 3.00, *α* = 1.50)	50	5	7	4	1	8	2	3	6
	80	3	7	4	1	8	2	5	6
	120	5	7	2	1	8	3	4	6
	200	4.5	6	2	1	8	3	4.5	7
	300	4	6	2	1	7.5	3	5	7.5
(*η* = 1.60, *σ* = 3.00, *α* = 1.50)	50	4	5	3	1	8	2	6	7
	80	4	6	2	1	7	3	5	8
	120	4	6	2	1	7	3	5	8
	200	4	5	2	1	7	3	6	8
	300	4	6	2	1	7	3	5	8
(*η* = 2.75, *σ* = 3.00, *α* = 1.50)	50	4	7	3	1	8	2	5	6
	80	4	7	2.5	1	8	2.5	5	6
	120	4	7	2	1	8	3	5	6
	200	4	6	2	1	8	3	5	7
	300	4	6	2	1	8	3	5	7
(*η* = 0.40, *σ* = 0.50, *α* = 4.00)	50	4	6	8	1	7	2	3	5
	80	5	6	7	1	8	2	3	4
	120	6	5	7	1	8	2	4	3
	200	4	5.5	7	1	8	3	5.5	2
	300	4	7	5	1	8	3	6	2
(*η* = 1.60, *σ* = 0.50, *α* = 4.00)	50	4	7	6	1	8	3	5	2
	80	4	7	5	1	8	3	6	2
	120	5	7	4	1	8	3	6	2
	200	5	6	4	1	7	3	8	2
	300	5	6	3	1	7	4	8	2
(*η* = 2.75, *σ* = 0.50, *α* = 4.00)	50	4.5	7	4.5	1	8	2	6	3
	80	5	7	4	1	8	3	6	2
	120	5	8	4	1	6	3	7	2
	200	5	7	3	1	8	4	6	2
	300	5	7	3	1	8	4	6	2
(*η* = 0.40, *σ* = 1.75, *α* = 4.00)	50	5	7	6	1	8	2	4	3
	80	6	7	5	1	8	3	4	2
	120	6	7	3	1	8	5	4	2
	200	6	8	2	1	7	4.5	4.5	3
	300	5.5	8	2	1	7	4	5.5	3
(*η* = 1.60, *σ* = 1.75, *α* = 4.00)	50	5	7	4	1	8	3	6	2
	80	5	6	4	1	8	3	7	2
	120	5	6	4	1	7.5	3	7.5	2
	200	5	8	3	1	7	4	6	2
	300	5	8	3	1	7	4	6	2
(*η* = 2.75, *σ* = 1.75, *α* = 4.00)	50	5	7	3.5	1	8	3.5	6	2
	80	5	8	3	1	7	4	6	2
	120	5	7	3	1	8	4	6	2
	200	5	8	3	1	7	4	6	2
	300	4	8	2	1	7	5	6	3
(*η* = 0.40, *σ* = 3.00, *α* = 4.00)	50	5	7	6	2	8	3	4	1
	80	5	7	6	1	8	3	4	2
	120	6	7	3	1	8	4	5	2
	200	4.5	8	3	1	7	4.5	6	2
	300	5	7	2	1	8	4	6	3
(*η* = 1.60, *σ* = 3.00, *α* = 4.00)	50	5	6	4	1	8	3	7	2
	80	5	6.5	3	1	8	4	6.5	2
	120	5	8	3	1	7	4	6	2
	200	5	6	3	1	8	4	7	2
	300	4	7	3	1	8	5	6	2
(*η* = 2.75, *σ* = 3.00, *α* = 4.00)	50	5	7	3	1	8	4	6	2
	80	5	7	3	1	8	4	6	2
	120	4	7	3	1	8	5	6	2
	200	5	7	3	1	8	4	6	2
	300	5	7	2	1	8	4	6	3
**∑ Ranks**		**592**	**868**	**471.5**	**142**	**998.5**	**394**	**719**	**675**
**Overall Rank**		**4**	**7**	**3**	**1**	**8**	**2**	**6**	**5**

## 9 Applications and numerical computations of VaR and TVaR

The key application of the heavy-tailed distributions is the insurance loss phenomena or extreme value theory. Here, we consider two data sets from insurance sciences. The flexibility of the NEHTW model was illustrated by analyzing two insurance data sets. Furthermore, corresponding to the analysed data sets, we calculate the VaR and TVaR of the NEHTW and Weibull models to show empirically, using the two real data sets, that NEHTW model has a heavier tail than, its sub-model, Weibull distribution.

The comparison of the NEHTW distribution is made with some important distributions including two-parameter Weibull, Lomax, Burr-XII (BX-II), exponentiated Lomax (EL) and beta Weibull (BW) distributions.

To decide about the best fitting among the competitive distributions, certain comparative tools called, AIC, BIC, Cramer Von Mises (CM), Anderson Darling (AD), and KS statistic with its corresponding *p*-value are taken in to account. The formulae of these discrimination measures can be explored in [30].

### 9.1 Applications to earthquake insurance and vehicle insurance losses data

In this subsection, we consider two practical insurance applications to show the flexibility of the NEHTW model in practice.

#### 9.1.1 The earthquake insurance data

**Fig 5:**
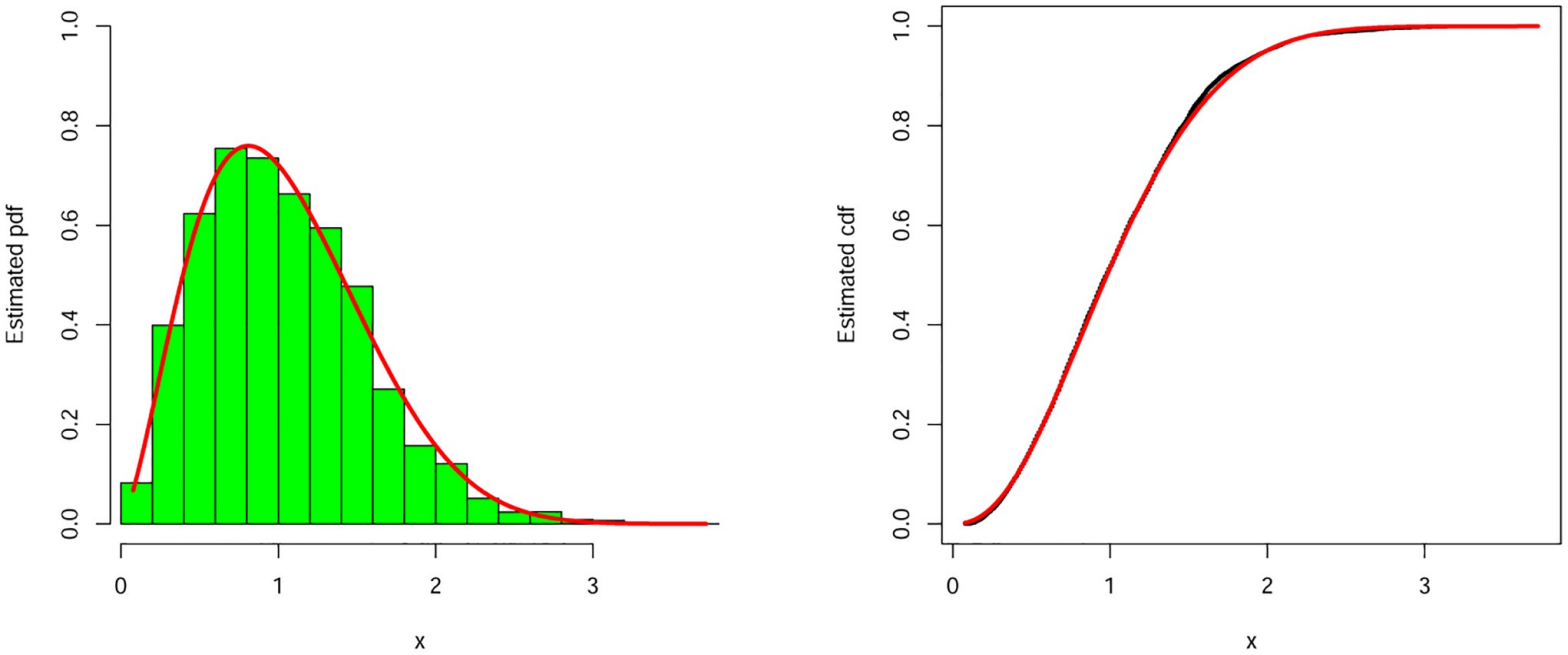
Estimated density and distribution functions of the NEHTW distribution for earthquake insurance data set.

**Fig 6:**
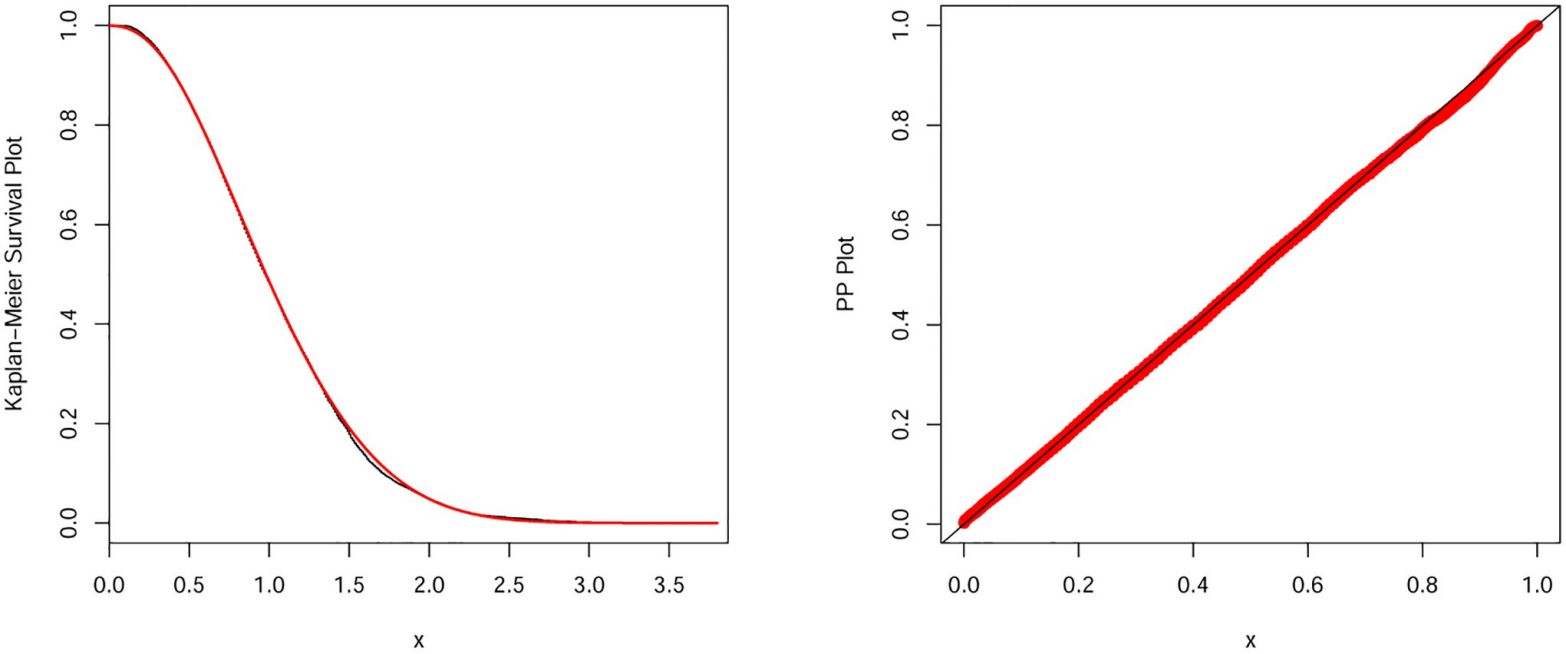
Kaplan Meier survival and PP-plots of the NEHTW distribution for earthquake insurance data set.

We begin with our first illustration by considering a heavy-tailed data set which refers to the earthquake insurances. This data set is available online at: https://earthquake.usgs.gov/earthquakes. The MLEs with standard errors (in parentheses) of the parameters and the estimated values of the considered measures were reported in Tables 9 and 10. Based on the values in Table 10, we conclude that the NEHTW model provide adequate fits as compared to other competitors. So, we prove empirically that NEHTW distribution can be a better model than other competitive models. Furthermore, corresponding to the earth quick insurance data, the estimated pdf and cdf of the NEHTW model were displayed in Fig 5. The Kaplan Meier survival (KMS) and PP plots are presented in Fig 6. The plots show that the NEHTW model has the best fitting to the earthquake insurance data.

**Table 9:** Parameters estimates and standard errors (in parentheses) of the NEHTW model with other models.

*Dist*.	$\hat{\alpha }$	$\hat{\gamma }$	$\hat{\eta }$	$\hat{\sigma }$	$\hat{a}$	$\hat{b}$
NEHTW	2.327 (0.03176)		0.5537 (0.01786)	1.5637 (0.0940)		
Weibull	2.1130 (0.01163)	0.7118 (0.00596)				
Lomax	73.690 (5.2704)	75.956 (5.4564)				
B-XII	1.219 (0.0092)	2.967 (0.0185)				
EL	49.238 (4.0760)	23.005 (1.9816)			4.4594 (0.0576)	
BW	1.7172 (0.03718)	1.256 (0.0877)			1.495 (0.0590)	0.807 (0.06793)

**Table 10:** Analytical measures of the NEHTW distribution and other competitors.

*Dist*.	AIC	BIC	AD	CM	K-S	p-value
NEHTW	27410.90	27435.75	5.119	0.490	0.0185	0.703
Weibull	27461.75	27475.60	8.495	1.078	0.0217	0.519
Lomax	40390.05	40404.19	10.062	5.139	0.235	0.478
B-XII	28662.28	28678.03	9.509	7.502	0.0623	0.221
EL	28062.61	28086.22	8.536	6.902	0.0459	0.337
BW	27450.49	27456.88	8.012	1.017	0.0197	0.578

#### 9.1.2 The vehicle insurance loss data

**Fig 7:**
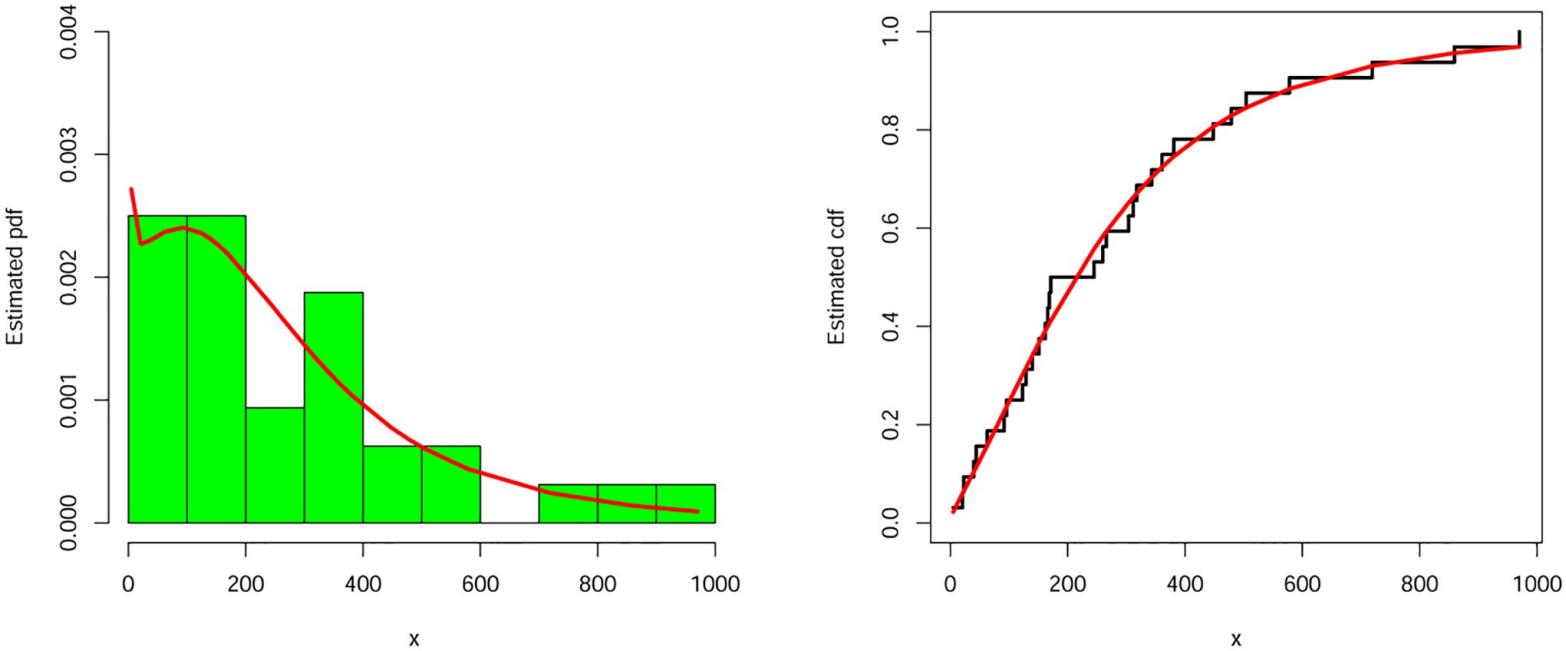
Estimated density and distribution functions of the NEHTW distribution for vehicle insurance losses data.

**Fig 8:**
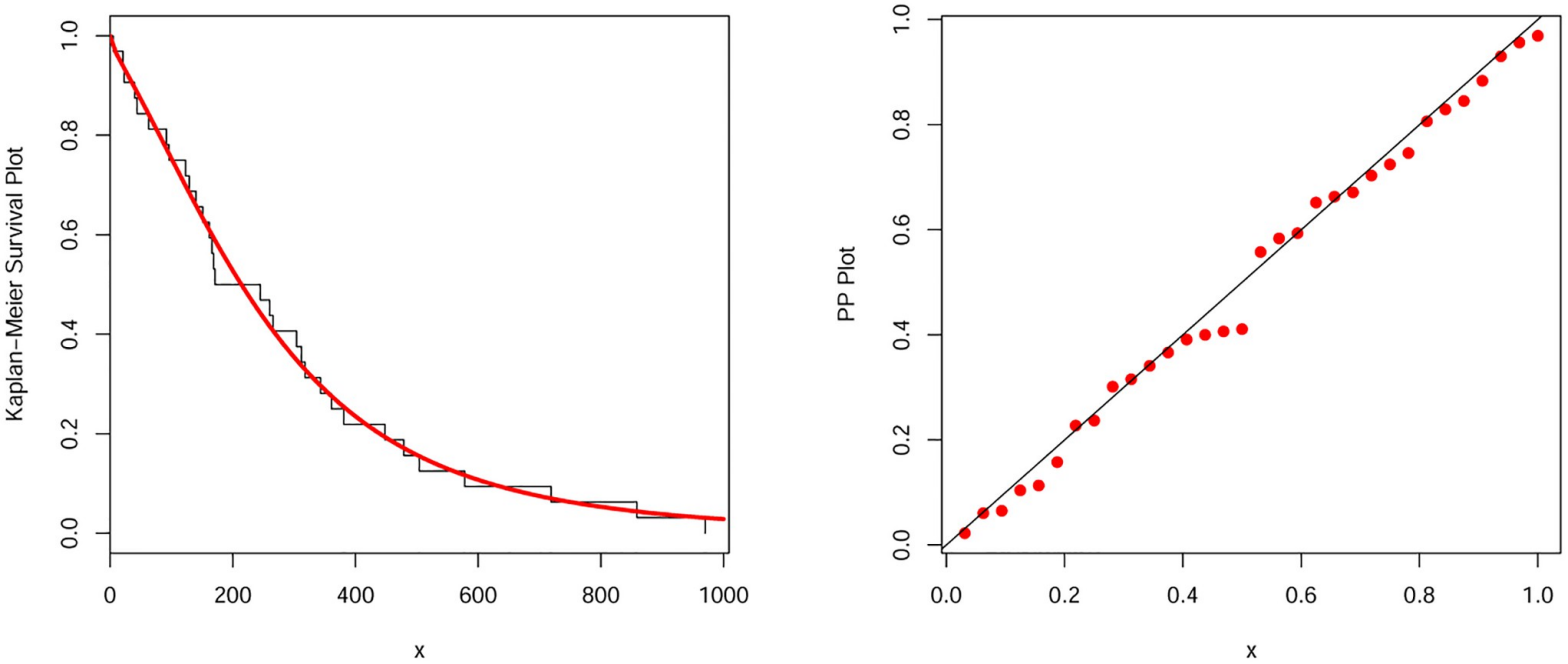
Kaplan Meier survival and PP-plots of the NEHTW distribution for vehicle insurance losses data.

The second data set is about vehicle insurance losses and it is available online at the following URL: https://data.world/datasets/insurance. The parameters estimates and associated standard error (in parentheses) of the NEHTW model and other models were listed in Table 11. The values of analytical measures for the NEHTW and other studied models were reported in Table 12. The values in Table 12 show that the NEHTW model outperforms all fitted models. The fitted pdf and cdf of the NEHTW model were shown in Fig 7. The PP plot and KMS plot of the NEHTW model were displayed in Fig 8. It is noted, from Fig 8, that the NEHTW model has a close fits to the PP and KMS plots.

**Table 11:** Parameters estimates and standard errors of the NEHTW model with other models.

*Dist*.	$\hat{\alpha }$	$\hat{\gamma }$	$\hat{\eta }$	$\hat{\sigma }$	$\hat{a}$	$\hat{b}$
NEHTW	0.304 (0.0297)		1.271 (0.8112)	0.004 (0.0020)		
Weibull	0.980 (0.0762)	0.7118 (0.0018)				
Lomax	1.355 (0.4334)	215.406 (89.618)				
B-XII	0.032 (0.1134)	6.022 (21.26711)				
EL	1.348 (0.4035)	89.857 (61.3795)			2.495 (0.9078)	
BW	1.184 (0.2250)	0.013 (0.0215)			0.856 (0.5287)	0.087 (0.1644)

**Table 12:** Analytical measures of the NEHTW distribution and other competitors.

*Dist*.	AIC	BIC	AD	CM	K-S	p-value
NEHTW	430.734	435.132	0.143	0.023	0.089	0.941
Weibull	439.909	442.902	0.223	0.033	0.103	0.868
Lomax	438.522	441.453	0.520	0.083	0.207	0.108
B-XII	503.451	506.383	1.362	0.228	0.416	0.098
EL	438.725	443.122	0.887	0.146	0.142	0.489
BW	431.574	437.437	4.588	0.792	0.087	0.949

### 9.2 The VaR and TVaR based on the two insurance data sets

**Fig 9:**
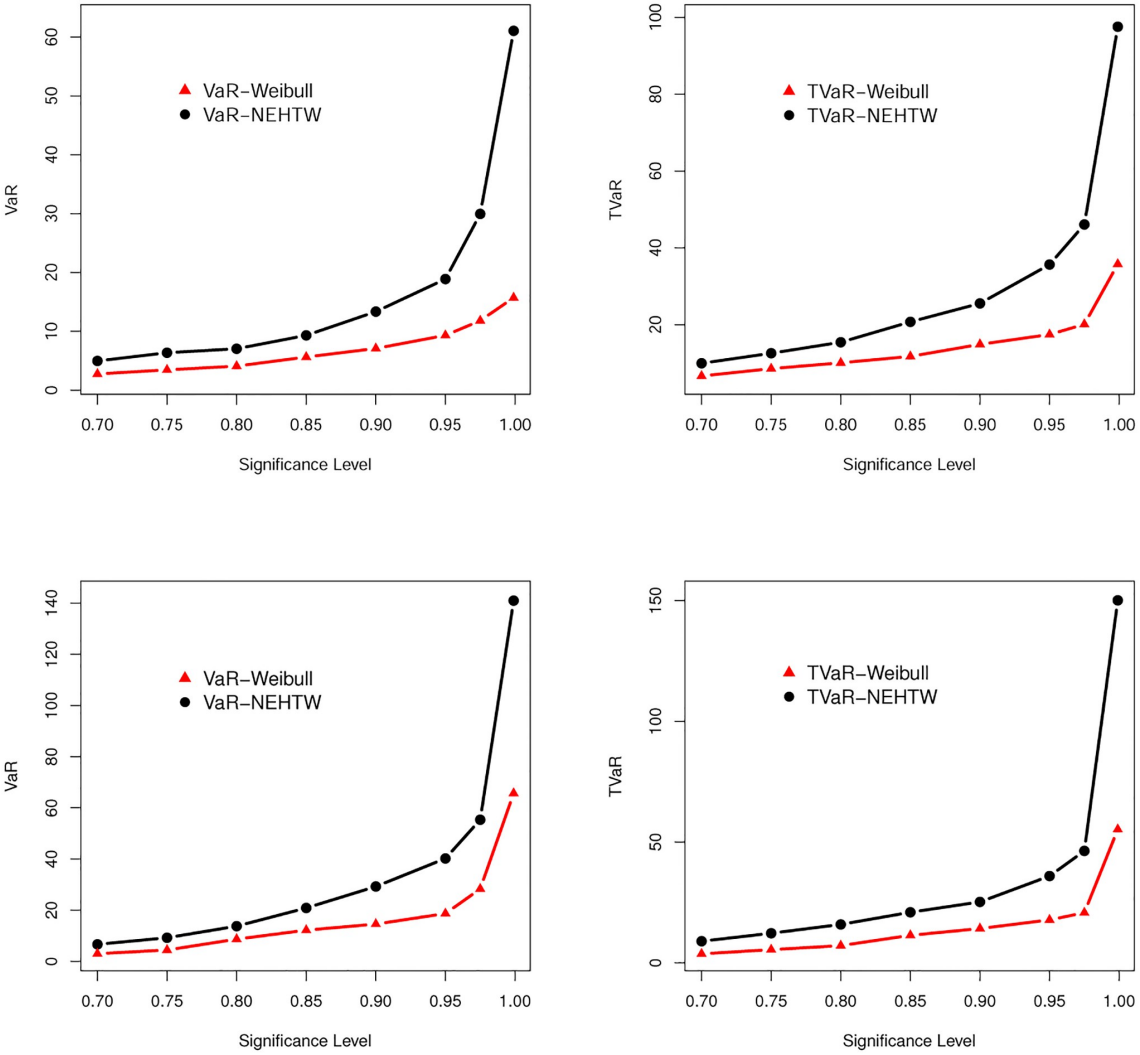
Graphical representation of the results provided in Tables 13 and 14.

This subsection is devoted to exploring the VaR and TVaR measures of the NEHTW and Weibull distributions via the estimated parameters of the two analyzed insurance data sets previously discussed in Section 7.1. The empirical results were listed in Tables 13 and 14 and they are displayed graphically in Fig 9.

**Table 13:** The values of VaR and TVaR for earthquake insurance data.

Dist.	Par	Significance Level	VaR	TVaR
Weibull	$\hat{\alpha }=2.1130$ $\hat{\gamma }=0.7118$	0.700	2.7654	6.6701
		0.750	3.4569	8.5826
		0.800	4.0956	10.0960
		0.850	5.6425	11.7678
		0.900	7.0943	14.9044
		0.950	9.3248	16.4693
		0.975	11.8211	19.1276
		0.999	15.7187	35.7890
NEHTW	$\hat{\alpha }=2.327$ $\hat{\eta }=0.5537$ $\hat{\sigma }=1.5637$	0.700	4.9763	9.9809
		0.750	6.3683	13.5909
		0.800	7.0445	18.4569
		0.850	9.3206	21.7643
		0.900	13.3567	25.5654
		0.950	18.8906	31.6730
		0.975	23.9543	36.0965
		0.999	61.0672	97.5673

**Table 14:** The values of VaR and TVaR for vehicle insurance losses data.

Dist.	Par	Significance Level	VaR	TVaR
Weibull	$\hat{\alpha }=0.980$ $\hat{\gamma }=0.711$	0.700	2.9876	3.6908
		0.750	4.4312	5.4321
		0.800	8.6784	7.0983
		0.850	12.1938	11.3799
		0.900	14.5644	14.1735
		0.950	18.6894	17.7588
		0.975	28.3251	20.7490
		0.999	65.6786	55.2345
NEHTW	$\hat{\alpha }=0.304$ $\hat{\eta }=1.271$ $\hat{\sigma }=0.004$	0.700	6.6543	8.9238
		0.750	9.2341	12.2304
		0.800	13.7544	15.8765
		0.850	20.8753	20.9245
		0.900	29.2534	25.2098
		0.950	40.2093	35.9213
		0.975	55.3428	46.3214
		0.999	140.9786	150.0789

The results for the two actuarial measures, Var and TVaR, of the NEHTW and Weibull distributions, which were reported in Tables 13 and 14, show that the NEHTW distribution has a heavier tail than the tail of Weibull distribution and hance it can be used for modelling heavy-tailed insurance data.

## 10 Conclusions

In this article, a new family of heavy-tailed distributions is proposed and studied. A special sub-model of the introduced family called, the new extended heavy-tailed Weibull (NEHTW) distribution is discussed in detail. The introduced NEHTW model is very flexible and can be adopted effectively to model data with heavy tails which encountered in insurance science. The maximum likelihood and other seven estimation approaches are adopted to estimate the NEHTW parameters. Their performances are assessed using detailed simulation results which are ranked to explore the best estimation approach for the model parameters. Based on our study, the maximum product of spacing is recommended to estimate the NEHTW parameters. Two important risk measures of the NEHTW model are studied numerically and empirically using a real-life insurance data. Both numerical and empirical studies of the two risk measures show that the introduced NEHTW distribution has heavier tail than its sub-model and can be used quite effectively in modelling heavy tailed insurance data sets. Finally, practical applications to insurance data sets are analyzed and the comparisons of the NEHTW model are made with some other important competitors. The practical applications and simulation of the two actuarial measures show that the NEHTW distribution is a good alternative for modelling heavy-tailed insurance data.

There are some possible future extensions to this study, such as a discrete version of the proposed family can be introduced following the work of [31]. Furthermore, a survival regression extended heavy-tailed Weibull model can be addressed for complete and censored data.
