# Correction: A new asymmetric extended family: Properties and estimation methods with actuarial applications

**DOI:** 10.1371/journal.pone.0315953

**Published:** 2024-12-12

**Authors:** Hassan M. Aljohani, Sarah A. Bandar, Hazem Al-Mofleh, Zubair Ahmad, M. El-Morshedy, Ahmed Z. Afify

There is an error in affiliation 4 for author Zubair Ahmad. The correct affiliation 4 is: Department of Statistics, Quaid-i-Azam University, Islamabad 45320, Pakistan.
